# Fluorocarbene, fluoroolefin, and fluorocarbyne complexes of Rh†
†Electronic supplementary information (ESI) available. CCDC 1518497–1518499. For ESI and crystallographic data in CIF or other electronic format see DOI: 10.1039/c6sc05391b


**DOI:** 10.1039/c6sc05391b

**Published:** 2017-02-16

**Authors:** Christopher J. Pell, Yanjun Zhu, Rafael Huacuja, David E. Herbert, Russell P. Hughes, Oleg V. Ozerov

**Affiliations:** a Department of Chemistry , Texas A&M University , College Station , TX 77842 , USA . Email: ozerov@chem.tamu.edu; b Department of Chemistry , Dartmouth College , Hanover , New Hampshire 03755 , USA

## Abstract

The manuscript reports the synthesis, characterization, and analysis of electronic structure in a series of complexes of small perfluorocarbon ligands with the (PNP)Rh fragment (where PNP is a diarylamido/bis(phosphine) pincer ligand). Reactions of (PNP)Rh(TBE) as the source of (PNP)Rh with CHF_3_ and C_2_HF_5_ produced perfluoroalkylidene complexes (PNP)Rh=CF_2_ and (PNP)Rh=C(F)(CF_3_). (PNP)Rh=CF_2_ could also be obtained *via* the reaction of (PNP)Rh(TBE) with Me_3_SiCF_3_/CsF, with an admixture of (PNP)Rh(C_2_F_4_), where TBE = *tert*-butylethylene. Abstraction of fluoride from these neutral (PNP)RhC_*x*_F_*y*_ complexes was successful, although only abstraction from (PNP)Rh=CF_2_ allowed unambiguous identification of the Rh product, [(PNP)Rh≡CF]^+^. DFT computational studies allowed comparison of relative energies of (PNP)Rh(C_2_F_4_) and [(PNP)Rh(C_2_F_3_)]^+^ isomers as well as comparisons between the electronic structure of the =CF_2_, C_2_F_4_, and ≡CF^+^ complexes and their hydrocarbon analogues.

## Abstract 2

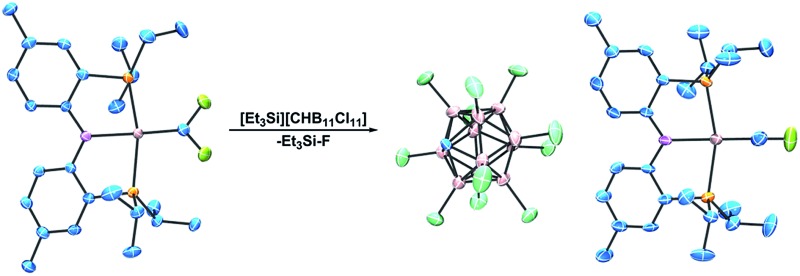
The manuscript reports a series of complexes of small perfluorocarbon ligands with the (PNP)Rh fragment, analysis of their electronic structure, and comparison to the hydrocarbon analogues and complexes of CO and NO^+^.

## Introduction

**Fig. 1:**
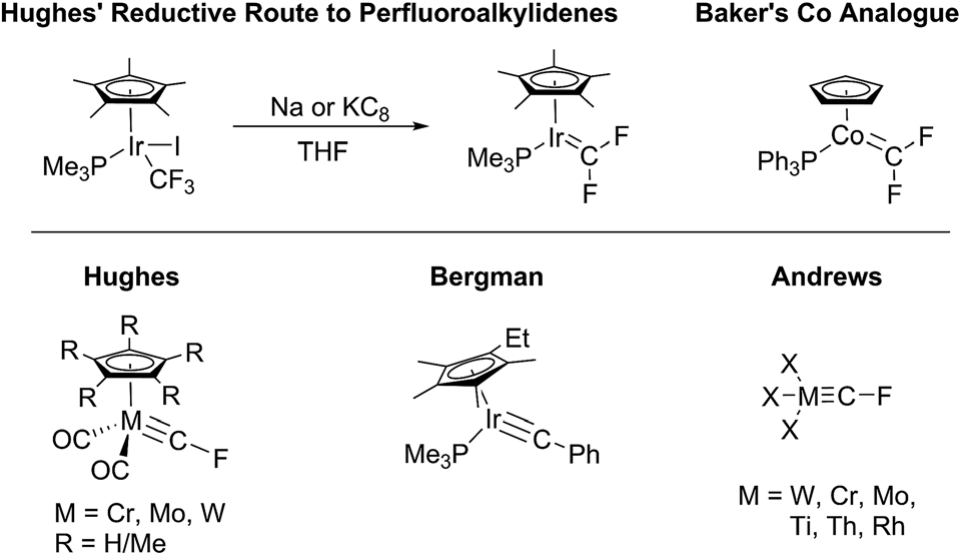
Perfluoroalkylidenes from Hughes and Baker. Isolated fluorocarbynes by Hughes, the Ir carbyne by Bergman, and matrix-trapped fluorocarbynes by Andrews.

Organofluorine chemistry's major impact on the world of industrial chemistry has inspired many investigations into the unique properties that are inherent to molecules and materials containing C–F bonds. Transition metal complexes containing perfluorocarbon ligands are an important subset of these studies since they exhibit distinctive bonding properties^1^ and can mediate perfluoroalkyl–carbon bond forming processes.^2^ Group 9 perfluoroalkylidenes have garnered interest in the past decade after Hughes developed a simple reductive method for making Ir=CFR complexes (Fig. 1, top) from iridium-fluoroalkyl precursors.^3^ These complexes have been analyzed in the context of their potential intermediacy in perfluorolefin metathesis,^4^ and more recently the Baker group has shown that analogous cobalt perfluorocarbenes (Fig. 1, top)^5^ are capable of undergoing a [2 + 1] cycloaddition with CF_2_ (ref. 6) and [2 + 2] cycloaddition with C_2_F_4_.^7^ Analogous chemistry was also reported for a difluorocarbene complex of Ni(0).^8^ Baker has also shown that cationic cobalt(iii) difluorocarbenes could undergo migratory insertion into perfluoroalkyl ligands, possibly providing a blueprint for transition metal catalyzed perfluoroolefin polymerization.^9^

The only family of isolable terminal fluoromethylidyne complexes known to date are the Cp*M(CO)_2_(CF) compounds (M = Cr, Mo, W) reported by Hughes and co-workers (Fig. 1, bottom).^10^ The Andrews group has reported a number of fluoromethylidyne complexes of the general formula X_3_M(CF) (Fig. 1, bottom; X = halogen) *via* trapping laser ablated metal atoms in argon/halocarbon matrices at *ca.* 10 K.^11^ Most of the isolable terminal carbyne complexes are complexes of metals of groups 6,^12,13^ 7,^13,14^ and 8.^15,16^ A few examples are known for group 5.^17^ In group 9, one 18-electron complex has been fully characterized for Ir by Bergman *et al.* (Fig. 1, bottom),^18^ and one square planar 16-electron complex was mentioned in passing for Rh by Werner *et al.*,^19^ as a component of a reaction mixture. The “concentration” of metal carbyne complexes in the middle of the transition metal series can be compared with similar trends for other metal-element multiple bonds.^20,21^ In this report, we describe the synthesis, characterization, and analysis of electronic structure of a rare cationic fluoromethylidyne complex of Rh, as well as related Rh perfluoroalkylidene complexes.

## Results and discussion

### Synthesis of CF_2_, C_2_F_4_, and CFCF_3_ complexes

We recently reported reactions of the (PNP)Rh fragment with aryl carboxylates, including aryl-oxygen oxidative addition.^22^ The (PNP)Rh acyl-oxygen oxidative addition product of phenyl trifluoroacetate, (PNP)Rh(C=OCF_3_)(OPh), could be thermolysed to produce (PNP)Rh(CO) and (PNP)Rh(CF_3_)(CO)(OPh) as major products. In that report, we noted that some other unidentified products were evident in trace amounts. We continued to be intrigued by one apparent trace product in particular that was consistently observed in 2–5% yield. For it, we observed a doublet of triplets both in the ^31^P{^1^H} NMR and ^19^F NMR spectra (coupling constants: ^1^ *J*_Rh–P_ = 146 Hz, ^2^ *J*_Rh–F_ = 49 Hz, ^3^ *J*_P–F_ = 30 Hz). These multiplicities implied a P_2_RhF_2_ NMR spin system – rather unexpected given the three fluorines in the CF_3_ group of the starting material. We noted that the ^19^F NMR chemical shift was itself uncommon (95.6 ppm) and in the range reported for various difluorocarbene complexes (*i.e.*, M=CF_2_).^5,10a,23,24^ The observed ^2^ *J*_Rh–F_ = 49 Hz was also similar to that of Grushin's *trans*-(Ph_3_P)_2_(F)Rh=CF_2,_ which possessed a ^2^ *J*_Rh–F_ of 33 Hz.^23^

**Scheme 1:**
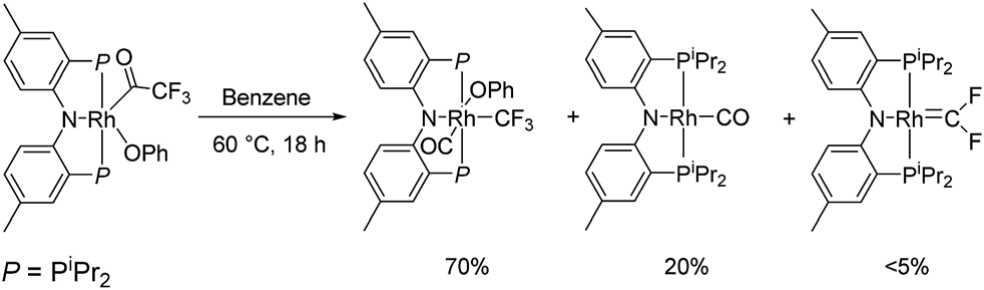
Initial observation of (PNP)Rh=CF_2_.

We hypothesized that this minor side product might be (PNP)Rh=CF_2_ and attempted an independent synthesis of it based on the procedure of Grushin *et al.* that yielded *trans*-(F)(PPh_3_)_2_Rh=CF_2_.^23^ Indeed, treatment of (PNP)Rh(TBE) (TBE = *tert*-butylethylene) with CsF/Me_3_SiCF_3_ (Ruppert's reagent) resulted in complete consumption of (PNP)Rh(TBE) and the formation of (PNP)Rh=CF_2_ and (PNP)Rh(C_2_F_4_) in about 85 : 15 ratio (NMR evidence).^25^ We were able to isolate (PNP)Rh=CF_2_ in 52% yield and of >98% purity by recrystallization. The ^31^P{^1^H} NMR and ^19^F NMR spectra of (PNP)Rh=CF_2_ obtained in this fashion were identical to that of the impurity we observed in the reaction in Scheme 1.

(PNP)Rh(TBE) showed no reaction with Me_3_SiCF_3_ alone. Similar to the other cases of use of CsF/Me_3_SiCF_3_, we propose that these reagents generate a CF_3_ anion equivalent that displaces TBE and then loses fluoride, resulting in the formal transfer of CF_2_ to Rh. Alternatively, CsF/Me_3_SiCF_3_ could be generating free CF_2_ which then binds to Rh. Using the CsF/Me_3_SiCF_3_ protocol, we could not avoid the formation of (PNP)Rh(C_2_F_4_) due to the generation of free C_2_F_4_ from the CsF/Me_3_SiCF_3_ mixture. C_2_F_4_ was observed by ^19^F NMR spectroscopy in control experiments where CsF and Me_3_SiCF_3_ were mixed in C_6_D_6_ and heated at 80 °C. No reaction was observed when (PNP)Rh=CF_2_ was treated with another equivalent of CsF/Me_3_SiCF_3_. This contrasts the reactivity of Baker's difluorocarbene cobalt(i) complexes^6^ which undergo a [2 + 1] cycloaddition with free CF_2_ to form cobalt tetrafluoroethylene complexes.

To date, we have not been able to formulate a reasonable proposal for how (PNP)Rh=CF_2_ could be formed from (PNP)Rh(COCF_3_)(OPh) (Scheme 1). The formation of M=CF_2_ by fluoride migration from M–CF_3_ is well precedented^26^ and is likely the key step in forming (PNP)Rh=CF_2_; the difficulty is with conceiving of a plausible fate of the other atoms of the original phenyl trifluoroacetate molecule.

**Scheme 2:**
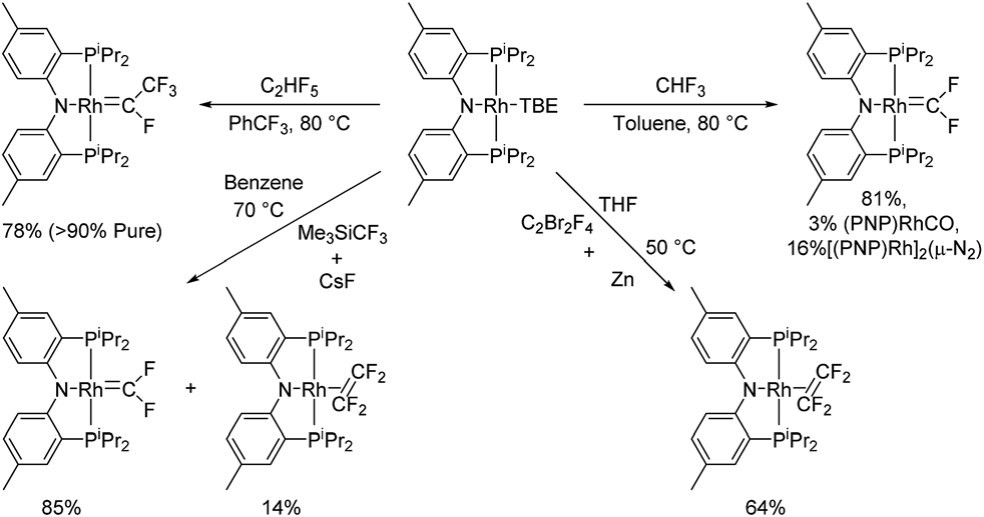
Synthesis of rhodium fluorocarbenes and tetrafluoroethylene complexes.

Goldman *et al.* documented formation of (PCP)Ir=CF_2_ in a reaction of a (PCP)Ir source with HCF_3_.^24^ This reaction proceeded *via* C–H oxidative addition of HCF_3_ to Ir followed by loss of HF. In a similar vein, we found that (PNP)Rh(TBE) reacted with HCF_3_ at 80 °C to provide a mixture of compounds containing (PNP)Rh=CF_2_ as a major product (>80%) with (PNP)Rh(CO) and [(PNP)Rh]_2_(μ-N_2_) as minor products (Scheme 2). Commercial HCF_3_ contains dinitrogen as an impurity. Hydrolysis of a difluorocarbene complex to a carbonyl complex has precedent,^27^ but attempts to purposefully hydrolyze (PNP)Rh=CF_2_ proved to be unsuccessful, reminiscent of Baker's cobalt fluorocarbene complexes.^5^ It is possible that hydrolysis of (PNP)Rh=CF_2_ only takes place in the presence of HF (a by-product of (PNP)Rh=CF_2_ generation). We observed no intermediates^28^ in the reaction of (PNP)Rh(TBE) with HCF_3_, which may indicate that dissociation of TBE^29^ is the rate-limiting step.

An analogous reaction of (PNP)Rh(TBE) with C_2_HF_5_ was attempted as a potential means to access (PNP)Rh(C_2_F_4_). However, the major product of this reaction turned out to be a tetrafluoroethylidene complex (PNP)Rh=C(F)(CF_3_) (Scheme 2). Dinitrogen impurity in C_2_HF_5_ led to the known^29^ [(PNP)Rh]_2_(μ-N_2_) as a major side product, whose content could be reduced by degassing C_2_HF_5_ using the “freeze–pump–thaw” technique. (PNP)Rh=CF_2_ was also observed as a side product composing 13% of the reaction mixture when (PNP)Rh(TBE) was treated with 2 atm of C_2_HF_5_ and heated overnight at 80 °C. (PNP)Rh=C(F)(CF_3_) could be isolated in >90% purity with (PNP)Rh(CO) composing the rest of the mixture. The synthesis of (PNP)Rh(C_2_F_4_) was instead accomplished by thermolysis of (PNP)Rh(TBE) in a solution containing C_2_F_4_ which was generated *in situ* by reducing C_2_F_4_Br_2_ with 1.5 eq. of Zn powder at 50 °C in THF (Scheme 2). (PNP)Rh(C_2_F_4_) was isolated in 64% yield as a pure solid.

The presence of multiple NMR-active nuclei provided for information-rich NMR spectra of (PNP)Rh=CF_2_, (PNP)Rh=C(F)(CF_3_), and (PNP)Rh(C_2_F_4_). All three complexes displayed *C*_2v_-symmetric NMR spectra in solution at ambient temperature. The carbene complexes (PNP)Rh=CF_2_ and (PNP)Rh=C(F)(CF_3_) displayed characteristic ^13^C NMR resonances at 206.3 and 225.0 ppm. In the CF_2_ complex the observation that the two fluorines couple identically to both phosphorus nuclei, and *vice versa*, is consistent with rapid rotation about the Rh=CF_2_ bond on the NMR timescale at room temperature. Likewise the observation of identical coupling of both P-nuclei to all four fluorines in the C_2_F_4_ complex is consistent with rapid rotation about the Rh–alkene bond axis. Small energy barriers to these rotations are calculated by DFT (see below).

**Fig. 2:**
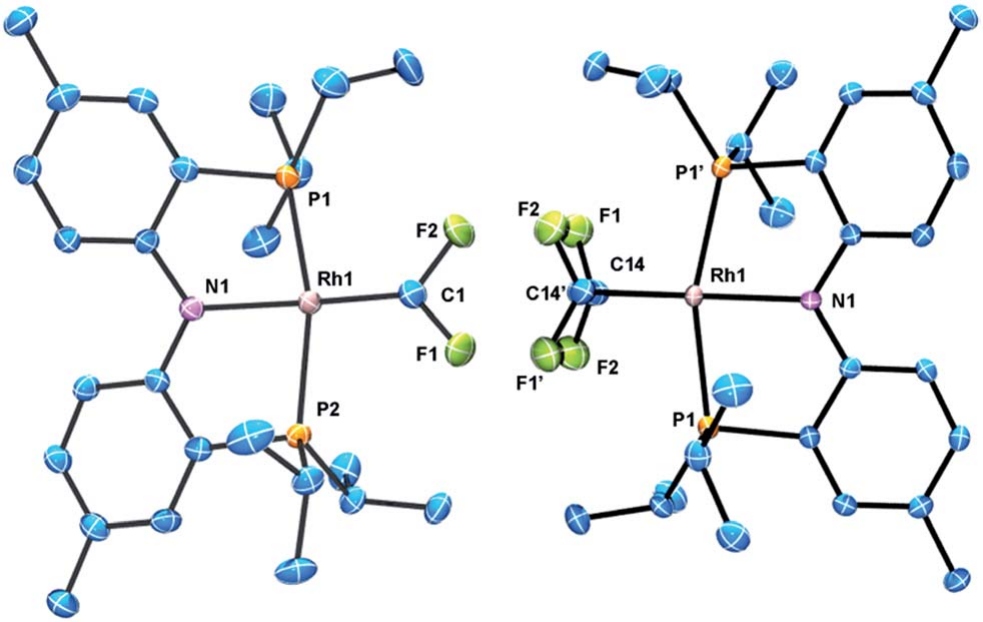
ORTEPs of (PNP)Rh=CF_2_ (left) and (PNP)Rh(C_2_F_4_) (right). The ellipsoids are set at the 50% probability level, and hydrogen atoms are omitted for clarity. Selected bond distances (Å) and angles (°) for (PNP)Rh=CF_2_: Rh1–C1, 1.821(4); Rh1–N1, 2.043(3); C1–F1, 1.335(4); C1–F2, 1.348(5); N1–Rh–C1, 171.39(15); F2–C1–F1, 100.8(3); Rh1–C1–F2, 130.1(3); Rh1–C1–F1, 128.6. (PNP)Rh(C_2_F_4_): Rh1–C14, 2.006(3); Rh1–N1, 2.054(3); C14–F1, 1.378(3); C14–F2, 1.361(3); C14–C14′, 1.354(7); C14–Rh–C14′, 39.4(2); C14–Rh–N1, 160.28(10).

The identity of (PNP)Rh=CF_2_ and (PNP)Rh(C_2_F_4_) was confirmed by X-ray diffraction studies on suitable single crystals (Fig. 2). Treating the CF_2_ or C_2_F_4_ ligands as occupying a sole coordination site, the coordination environment about Rh is approximately square planar in both molecules. The CF_2_ unit in (PNP)Rh=CF_2_ lies approximately in that plane, while the C–C vector of the C_2_F_4_ ligand in (PNP)Rh(C_2_F_4_) is approximately perpendicular to it. The CF_2_ and C_2_F_4_ ligands evidently exert similar *trans*-influence as the Rh–N distances in (PNP)Rh=CF_2_ and (PNP)Rh(C_2_F_4_) are only different by *ca.* 0.01 Å. In general, the metrics of the Rh=CF_2_ unit in (PNP)Rh=CF_2_ are very similar to the Rh=CF_2_ unit in *trans*-(Ph_3_P)_2_(F)Rh=CF_2_. The structures of (PNP)Rh=CF_2_ and (PNP)Rh(C_2_F_4_) contain some close C–F···H contacts (C–F···H distances of 2.33–2.45) (F···C distances of 3.1–3.3 Å). While they are probably unavoidable in these molecules, these distances are short enough to be considered weak F···H interactions.^30^ C–F···H interactions have been observed in pincer-ligated zirconium complexes bearing a trifluoromethyl as a pendant group, which have also exhibited through-space H–F coupling visible in their ^1^H NMR spectra.^31^ However (PNP)Rh(C_2_F_4_) and (PNP)Rh=CF_2_ showed no through-space ^19^F–^1^H coupling to the isopropyl arms.

### Synthesis of cationic fluoromethylidyne

With compounds (PNP)Rh=CF_2_, (PNP)Rh(=CFCF_3_), and (PNP)Rh(C_2_F_4_) in hand, we contemplated whether one of the fluorides could be removed to yield cationic C_*x*_F_*y*_ complexes. Hughes *et al.* previously demonstrated proton-induced loss of fluoride from α-positions of Ir perfluoroalkyls,^3*a*^ and Baker recently demonstrated a Lewis-acid abstraction of a fluoride from N-heterocyclic fluoroalkenes to yield polyfluoroalkenyl imidazolium salts.^32^ There is significant precedent for electrophilic abstraction of an anionic heteroatom substituent from late-metal carbene complexes by a Lewis acid.^18,33^ Trialkylsilylium cations, in the form of their salts with halogenated carborane anions, are powerful Lewis acids with high affinity for fluoride.^34^ We and others have exploited them in catalytic C–F activation reactions^35^ and thus a [R_3_Si]^+^ reagent appeared perfect for fluoride abstraction.

Reactions of (PNP)Rh=CF_2_, (PNP)Rh(C_2_F_4_), and (PNP)Rh=C(F)(CF_3_) with [Et_3_Si–H–SiEt_3_][HCB_11_Cl_11_]^36^ or [(Et_3_Si)_2_OTf][HCB_11_Cl_11_]^37^ all generated the Et_3_SiF by-product, indicating that fluoride abstraction took place in all three cases. However, reactions of (PNP)Rh(C_2_F_4_) and (PNP)Rh=C(F)(CF_3_) resulted in mixtures of several products as seen by ^19^F NMR spectroscopy and typically broad or no signals were observed by ^31^P{^1^H} NMR spectroscopy. The reaction mixtures produced from the reaction of (PNP)Rh(C_2_F_4_) or of (PNP)Rh=C(F)(CF_3_) with [(Et_3_Si)_2_OTf][HCB_11_Cl_11_] did regenerate the corresponding starting material when treated with CsF. This indicates that fluoride abstraction from these two isomeric complexes generates isomers of [(PNP)Rh(C_2_F_3_)]^+^ that do not interconvert on the experimental time scale. Although we were not able to identify these compounds experimentally, DFT computational studies were used to investigated possible structures of the [(PNP)Rh(C_2_F_3_)]^+^ isomers (*vide infra*).

**Scheme 3:**
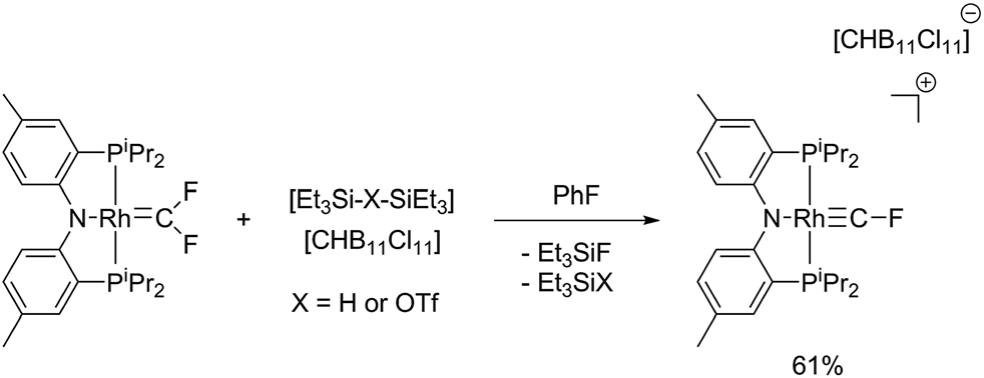
Synthesis of [(PNP)Rh≡CF][CHB_11_Cl_11_] *via* fluoride abstraction from (PNP)Rh=CF_2_.

On the other hand, reaction of (PNP)Rh=CF_2_ with [Et_3_Si–H–SiEt_3_][HCB_11_Cl_11_] cleanly and reproducibly generated a new Rh complex that displayed a P_2_RhF NMR spin system (Scheme 3). The key NMR spectroscopic features of this compound were the unusual ^19^F NMR chemical shift (66.2 ppm), the very high ^1^ *J*_C–F_ coupling constant of 470 Hz,^38^ and the rather substantial ^2^ *J*_Rh–F_ = 136 Hz.

These spectroscopic data are similar to those exhibited by Cp*(CO)_2_Mo≡CF, whose ^19^F NMR spectrum contained a singlet at 78.15 ppm, with a large ^1^ *J*_C–F_ coupling constant of 556 Hz evident by ^13^C NMR spectroscopy.^10*a*^ Hughes's other Cp(CO)_2_M≡CF (M = Cr, W) complexes also exhibited ^19^F NMR chemical shifts in this region with high *J*_C–F_ coupling constants.^10*b*^ Due to limited solubility in non-interactive solvents and the extensive coupling inherent to the fluoromethylidyne ^13^C NMR resonance in [(PNP)Rh≡CF]^+^, it was not observed by ^13^C{^31^P}, ^13^C{^1^H}, nor ^13^C{^19^F} NMR spectroscopy.

**Fig. 3:**
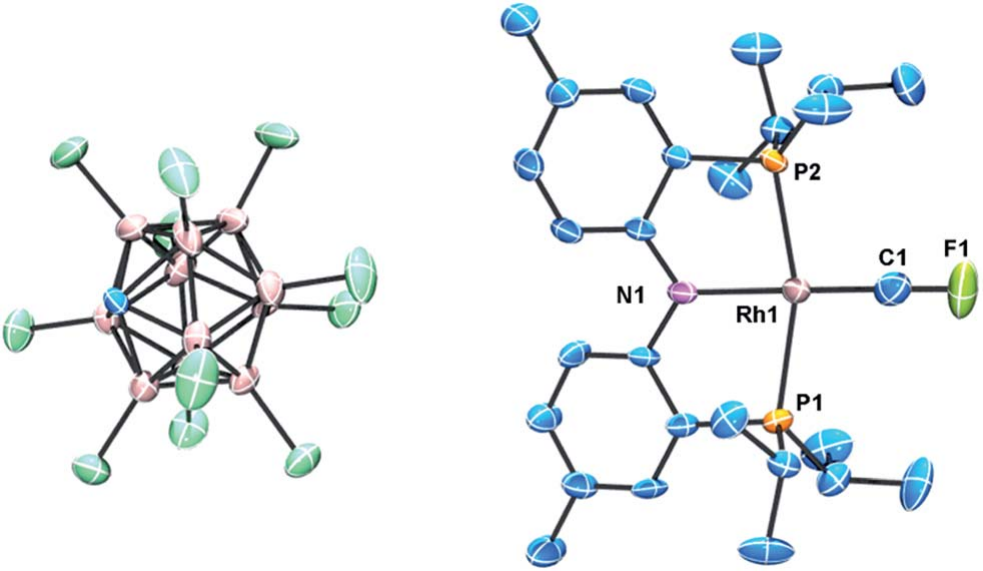
ORTEP of [(PNP)Rh≡CF][HCB_11_Cl_11_]. The ellipsoids are set at the 50% probability level, and hydrogen atoms are omitted for clarity. Selected bond distances (Å) and angles (°): Rh1–C1, 1.702(7); Rh1–N1, 2.019(4); C1–F1, 1.257(8); C1–Rh1–N1, 174.1(3); F1–C1–Rh1, 173.4(7).

X-ray quality crystals of [(PNP)Rh≡CF][CHB_11_Cl_11_] were studied using X-ray diffraction to yield a structure fully supportive of a fluorocarbyne formulation (Fig. 3). The structural and NMR spectroscopic features of [(PNP)Rh≡CF]^+^ are best reviewed in comparison with Cl_3_Rh≡CF and a few other relevant compounds. Andrews *et al.* observed IR spectroscopic evidence for Cl_3_Rh≡CF in reactions of laser-ablated rhodium atoms with CFCl_3_. A DFT calculation of this product predicted a Rh–C bond length of 1.740 Å and a Rh–C–F bond angle of 143.4°.^11*d*^ This compares with our observed Rh–C bond length of 1.702(7) Å and a Rh–C–F bond angle of 173.46°. Although both [(PNP)Rh≡CF]^+^ and Cl_3_Rh≡CF are four-coordinate, they contain different numbers of valence electrons: from a hypothetical point of view of a [CF]^+^ ligand, it is attached to a d^8^ Rh center in [(PNP)Rh≡CF]^+^, but to a d^7^ [Cl_3_Rh]^–^ fragment in Cl_3_Rh≡CF. The geometry of the RhCF unit in [(PNP)Rh≡CF]^+^ is similar to Bergman's iridium carbyne complex (Scheme 1), which possesses an Ir–C bond length of 1.734(6) Å and an Ir–C–C bond angle of 175.7(4).^18^ The Rh–C distance in [(PNP)Rh≡CF]^+^ is *ca.* 0.12 Å shorter than that in (PNP)Rh=CF_2_, consistent with the increase in the Rh–C bond order. The Rh–C bond distance in [(PNP)Rh≡CF]^+^ is also *ca.* 0.07 Å shorter than that in Werner's *trans*-(P^i^Pr_3_)_2_ClRh=C=C(Me)(H) square planar vinylidene complex.^39^

## Computational studies and discussion

### DFT structural studies

Modern Density Functional Theory (DFT) is a powerful tool with which to examine electronic structures and bonding trends in organometallic compounds.^40,41^ In addition, application of Natural Bond Orbital (NBO)^42–47^ methods allows insight into some of the subtleties of metal–ligand bonding.^42,44,46^ The NBO analysis also generates Wiberg Bond Indices (WBI),^48^ determined within the natural atomic orbital basis, providing one means of estimating bond orders between atoms. Trends in WBI values are also useful in tracking variations in bond multiplicities. The newly synthesized family of fluorocarbon complexes (PNP)Rh(C_2_F_4_), (PNP)Rh=CF_2_, and [(PNP)Rh≡CF]^+^ prompted a computational comparison with their (hypothetical) hydrocarbon analogues (PNP)Rh(C_2_H_4_), (PNP)Rh=CH_2_ and [(PNP)Rh≡CH]^+^ in order to assess the effects of fluorination on the metal–carbon bonding, and, for the carbene and carbyne complexes, to probe the nature and extent of the multiple bonding between the metal and carbon. In addition, since the CF^+^ ligand is isoelectronic with the well-known ligands NO^+^ and CO, it was of interest to establish trends in metal ligand and intraligand bonding between [(PNP)Rh≡CF]^+^, [(PNP)Rh(NO)]^+^, and (PNP)Rh(CO). Full molecule DFT studies were performed using the M06 functional^49,50^ and the triple-*ζ* LACV3P**++ basis set, which uses extended core potentials^51–54^ on heavy atoms and a 6-311G**++ basis^55–58^ for other atoms, as implemented in the Jaguar^59,60^ suite of programs. Full details are available as ESI.†

Selected bond lengths and computed WBI values for the calculated complexes are provided in Table 1, with metric comparisons to the available crystallographic structures reported here. The DFT calculated metrics are in good agreement with crystallographic numbers, giving confidence in the DFT metrics for the unknown complexes. One exception appears in (PNP)Rh(C_2_F_4_) in which the C–C distance for the coordinated alkene (1.354 Å) is only slightly longer than that in C_2_F_4_ itself (1.318 Å)^61^ and is much shorter than all other transition metal complexes of this perfluoroalkene in the Cambridge Structure Database.^62^ In contrast, the DFT calculated value (1.416 Å) is in good agreement with other crystallographically determined values^62^ and is much more sensible with respect to calculated WBI values (see below).

**Table 1:** Calculated and crystallographic bond lengths (Å)^*a*^ and Wiberg Bond Indices^*b*^ (WBI)

Compound	Rh–P_ave_	Rh–N	Rh–C_ave_	C–C	C–X_ave_ (ligand)
(PNP)Rh(C_2_H_4_)	2.332	2.098	2.165	1.394	1.088
	*0.462*	*0.378*	*0.453*	*1.474*	*0.930*
(PNP)Rh(C_2_F_4_)	2.378	2.100	2.048	1.416	1.346
	**2.3309(11)**	**2.054(3)**	**2.006(3)**	**1.354(7)** ^*c*^	**1.369(3)**
	*0.436*	*0.352*	*0.594*	*1.186*	*0.858*
(PNP)Rh=CH_2_	2.338	2.216	1.850	—	1.104
	*0.476*	*0.225*	*1.250*		*0.955*
(PNP)Rh=CF_2_	2.331	2.156	1.864	—	1.325
	**2.302(12)**	**2.043(3)**	**1.821(4)**		**1.341(5)**
	*0.462*	*0.297*	*1.168*		*0.911*
[(PNP)Rh≡CH]^+^	2.378	2.075	1.728	—	1.110
	*0.440*	*0.332*	*1.714*		*0.942*
[(PNP)Rh≡CF]^+^	2.384	2.061	1.740	—	1.247
	**2.337(16)**	**2.019(4)**	**1.702(7)**		**1.257(8)**
	*0.432*	*0.341*	*1.587*		*1.065*
[(PNP)Rh≡C–CF_3_]^+^	2.384	2.054	1.734	—	1.499
	*0.453*	*0.330*	*1.770*		*0.960*
(PNP)Rh(CO)	2.336	2.117	1.855	—	1.152
	*0.462*	*0.291*	*1.041*		*2.031*
[(PNP)Rh(NO)]^+^	2.393	2.019	1.778 (Rh–N)	—	1.141 (NO)
	*0.439*	*0.445*	*1.151*		*1.917*

^*a*^DFT calculated (M06/LACV3P**++) values are in plain text; X-ray crystallographic values are in bold.

^*b*^WBI values are in italics.

^*c*^This crystallographic value is questionable. See discussion in the text.

All the complexes examined here can be formally viewed as square planar d^8^ compounds, *i.e.*, as complexes of a d^8^, three-coordinate fragment (PNP)Rh with neutral or cationic ligands. The NBO perspective of bonding interactions in such compounds^42,46^ requires 4-electron/3-center bonds between the pair of *trans* ligands such that the alkene, carbene, and carbyne ligands of interest are always involved in a shared bonding interaction with the N of the PNP pincer. Clearly contributions to this shared interaction may be weighted differently in each case, and the WBI values should reflect this.

### Comparison of C_2_H_4_ and C_2_F_4_ ligands

**Fig. 4:**
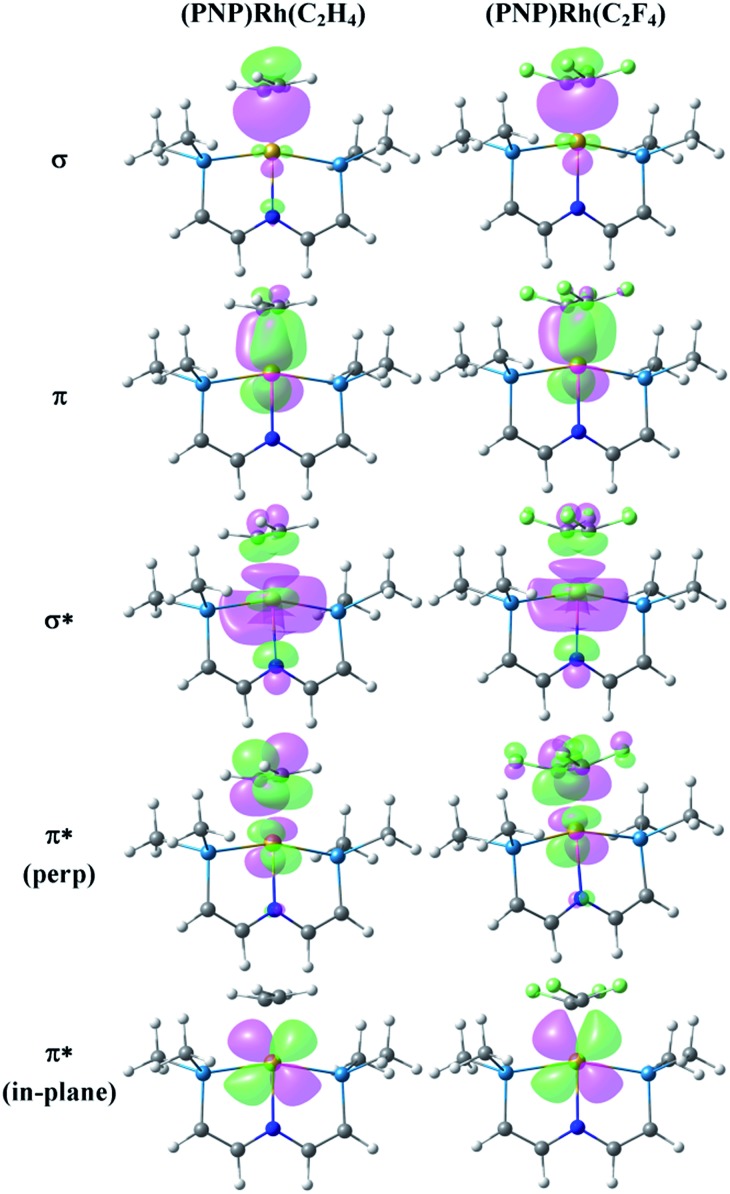
NLMOs for the bonding interactions between C_2_H_4_ (column 1) and C_2_F_4_ (column 2) and the (PNP)Rh fragment. For clarity the P^i^Pr_2_ groups have been replaced by PMe_2_ groups and the aryl part of the pincer truncated to P–CH=CH–N linkers.

The bonding between alkenes and transition metal fragments is well understood,^63^ but a comparison between C_2_H_4_ and C_2_F_4_ coordinated to identical metal–ligand fragments is rare. A classic intramolecular example involves CpRh(C_2_H_4_)(C_2_F_4_)^64^ but the hydrocarbon and fluorocarbon alkenes are necessarily bound to different fragments in this molecule. Fig. 4 illustrates the key Natural Localized Molecular Orbitals (NLMOs)^42,45,46^ arising from NBO calculations of interactions between C_2_H_4_ and C_2_F_4_ and the truncated^65^ (PNP)Rh fragment. The bonding orbitals (σ and π) are essentially localized on Rh and the alkene ligand, while the corresponding antibonding NLMOs show significant “tailing” involving the σ and π orbitals on the *trans*-N of the pincer ligand. This “tailing” is indicative of delocalization of these N electrons into the corresponding σ* and π* components of the Rh–alkene interaction; it is significantly greater for the σ* component and corresponds to the 3-center/4-electron bonding expected between *trans*-ligands in a d^8^ Rh(i) complex. The WBI values indicate significantly greater reduction in C–C bonding and increase in Rh–C bonding in coordinated C_2_F_4_ than in C_2_H_4_. This is consistent with the shorter Rh–C distances and with the idea of a more metallacyclopropane structure and stronger Rh–C bonding for the fluorinated alkene complex. Not surprisingly, stronger bonding to the fluorinated alkene results in weaker bonding to the *trans* ligand, with correspondingly lower WBI values for the Rh–N bonds (Table 1). In the π*-perp NMLO for the C_2_F_4_ there is also evidence for delocalization from F-lone pairs (see Fig. 4).

There are two Rh→alkene backbonding options involving π*-perp or π*-in-plane. Clearly the ground state conformation of the C_2_F_4_ complex utilizes the former, but the latter is available for an in-plane C_2_F_4_ conformation, leading to a low barrier for C_2_F_4_ rotation. Similar arguments for facile rotation of C_2_F_4_ ligands in Ru(ii) complexes have been put forth elsewhere.^66^ The free energy profile for C_2_F_4_ rotation was calculated using a truncated^65^ version of the PNP ligand (identical to that shown in Fig. 4), and is unusual. Relative to the perpendicular conformation observed in the ground state, two transition states were located. The first, lying 7.1 kcal mol^–1^ above the ground state, corresponds to a 45 degree rotation about the Rh–alkene bond axis, and the second, lying 8.7 kcal mol^–1^ above the ground state, is the conformation in which the fluoroalkene lies in the coordination plane. These barriers are low enough in energy that rotation should be fast on the NMR timescale, consistent with the observed NMR data. The barriers contrast with those for the corresponding C_2_H_4_ analogue, for which the in-plane conformation is a minimum, lying only 0.4 kcal mol^–1^ above the ground state, and the 45 degree conformation is a transition state lying 2.5 kcal mol^–1^ above the ground state.

### Comparison of CH_2_ and CF_2_ ligands

**Fig. 5:**
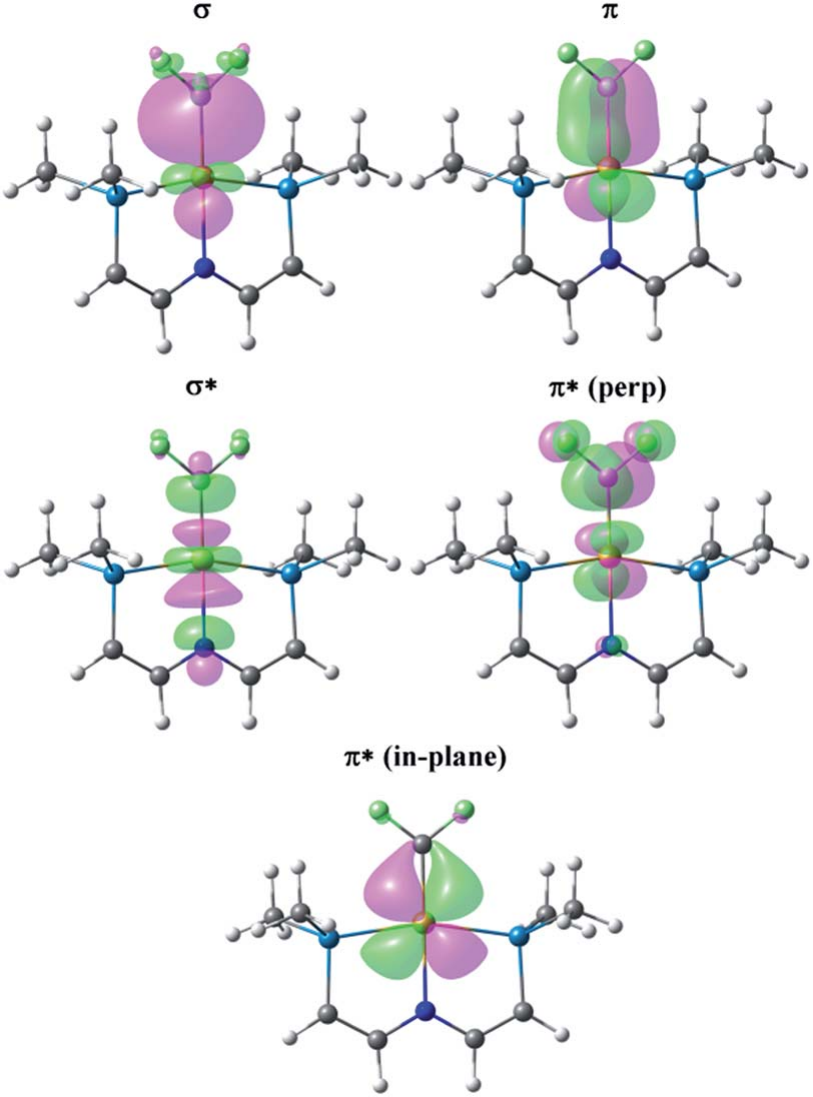
NLMOs for the bonding interactions between the CF_2_ ligand and the (PNP)Rh fragment. For clarity the P^i^Pr_2_ groups have been replaced by PMe_2_ groups and the aryl part of the pincer truncated to P–CH=CH–N linkers.

Fig. 5 presents the corresponding NLMOs for the CF_2_ complex with a truncated^65^ PNP ligand. Those for the CH_2_ analogue are very similar, except for the fluorine delocalizations into the π*-NLMO, and are not illustrated here but can be found in the ESI (Fig. S17†). The σ and π NLMOs are consistent with a formal double bond between Rh and the CF_2_ (or CH_2_) ligands, with the p-orbital on C and d-orbital on Rh providing the π-component. These NLMOs look essentially identical to those in Fig. 4, except for a more significant delocalization of the fluorine lone pairs in the π*-perp NLMO. But now there is competition between the rhodium d-orbital and the fluorine lone pair p-orbitals for π-bonding with the carbene carbon, as expected; in (PNP)Rh=CH_2_ only the metal can provide this π-bonding. Consequently π-bonding with fluorines diminishes π-bonding with Rh and, relative to the CH_2_ complex, the Rh–C WBI decreases significantly and the Rh–C distance increases; notably the C–F WBI is greater than in the previously discussed C_2_F_4_ complex. In contrast to the alkene ligands discussed above there is overall weaker metal–carbon bonding to CF_2_ than CH_2_ and the corresponding *trans*-Rh–N WBI value is larger for (PNP)Rh=CF_2_.

Facile CF_2_ ligand rotation is expected due to the availability of the π*-perp and π*-in-plane interactions. Calculations on the truncated^65^ ligand analogue of the CF_2_ complex reveal the same trend in conformational energetics observed for the C_2_F_4_ complex (*vide supra*). The in-plane CF_2_ conformation, with the π*-perp interaction, is the ground state, with two transition states at 45 degree and 90 degree (perpendicular to the coordination plane) lying 2.0 and 4.8 kcal mol^–1^ higher in energy, respectively. The barriers are consistent with the experimental observation of fast rotation on the NMR timescale. In contrast, the rotation of the corresponding CH_2_ ligand is even more facile, with the perpendicular, 45 degree and in-plane conformations lying at essentially equal energies.

### Comparison of CH^+^, CF^+^ and CCF_3_^+^ ligands

**Fig. 6:**
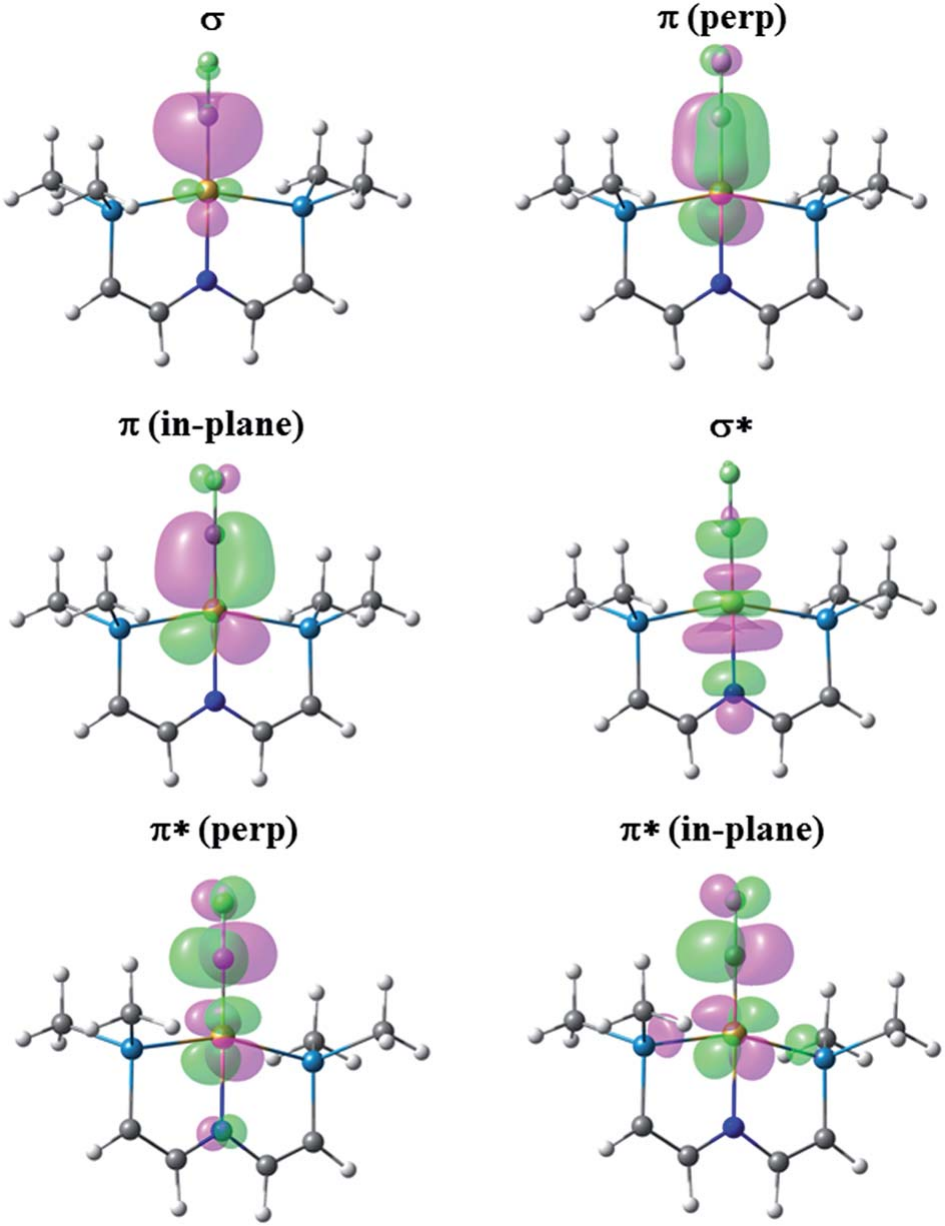
NLMOs for the bonding interactions between the CF^+^ ligand and the (PNP)Rh fragment. For clarity the P^i^Pr_2_ groups have been replaced by PMe_2_ groups and the aryl part of the pincer truncated to P–CH=CH–N linkers.

Fig. 6 presents the corresponding NLMOs for [(PNP)Rh≡CF]^+^. As with the carbene complexes, those for [(PNP)Rh≡CH]^+^ are similar except for enhanced “tailing” in the antibonding NLMOs for the CF^+^ complex. In contrast to the alkene and carbene complexes (*vide supra*) there is now a second fully engaged π-component for the Rh–C bond involving the in-plane d-orbital and a second p-orbital on the CF^+^ (or CH^+^) ligand. In the antibonding NLMOs we see the expected σ-donation from the *trans*-N in σ*, a small π-donation from *trans*-N in π*(perp), and a small donation from the Rh–P bonds in π*(in-plane). But once again the largest delocalizations in the π* NLMOs comes from the F lone pairs, interactions that cannot occur in [(PNP)Rh≡CH]^+^. Consequently the Rh–C WBI for [(PNP)Rh≡CH]^+^ is substantially larger than that for the CF^+^ analogue, consistent with the shorter Rh–C distance in the former; as before, a smaller Rh–C WBI in [(PNP)Rh≡CF]^+^ leads to a larger WBI for the *trans*-Rh–N bond. In the CCF_3_ analogue, in which F lone pair participation with the carbyne carbon is removed, a larger Rh–C WBI is calculated, with a correspondingly smaller Rh–N WBI.

### Degree of Rh–C multiple bonding

The Rh–C bonding in these carbene and carbyne complexes is inextricably linked to interactions with the *trans*-N since there are necessarily shared bonding components between *trans*-ligands, with additional complexities introduced by the fluorine substituents on carbon. So we cannot expect the Rh=CX_2_ interaction to be a true double bond, or that in the cationic Rh≡CX to be a triple bond, even though we may draw resonance structures that reflect these prejudices. However, the WBI values for both Rh=CX_2_ bonds (CH_2_ *1.250*; CF_2_ *1.168*) are significantly larger than unity, though not close to two, while those for the Rh≡CX cations are significantly larger still (CH *1.714*; CF *1.587*; CCF_3_ *1.770*), though not close to the bond order of three. Clearly there is significant multiple bonding between Rh and these unsaturated carbon ligands with higher bond orders to these ligands being reflected in lower bond orders to the *trans*-N.

### Comparison of CF^+^, NO^+^, and CO ligands

**Fig. 7:**
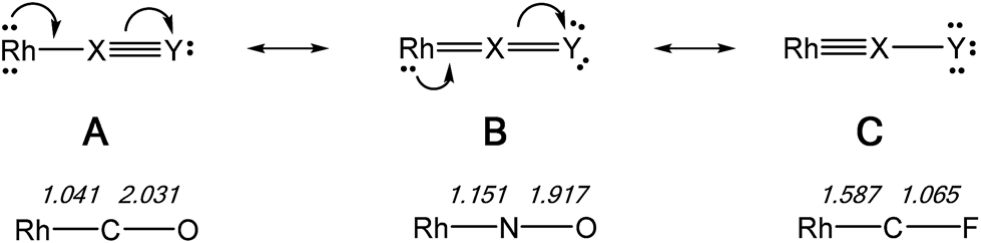
Resonance forms for the π-system in a linear Rh–X–Y ligand array, with WBI values for the bonds in Rh–C–O, Rh–N–O, and Rh–C–F complexes. All three complexes are isoelectronic and no formal charges are shown.

The NLMOs for the CO and NO^+^ complexes are similar to those of the CF^+^ compounds discussed previously and are not shown here. Considering this series of isoelectronic complexes as involving a linear Rh–X–Y array the three resonance forms (A, B, C) for the contiguous π-system are shown in Fig. 7, along with the WBI values for the appropriate bonds in the Rh–C–O, Rh–N–O and Rh–C–F complexes. The WBI values are consistent with progressively increased contributions of resonance forms C > B > A on changing the ligand from CO to NO^+^ to CF^+^, as expected from their relative π-acceptor abilities. Similar conclusions were reached for the fragments M(CO)_2_(XY) [M = Cr, Mo, W; XY = CO, NO^+^, CF^+^] in a previous study.^10*b*^

### Relative energies of isomeric fluorocarbon ligands

It was of interest to compare the relative energies of the (PNP)Rh(C_2_F_4_) complex with its carbene isomer (PNP)Rh(CFCF_3_). At the DFT/M06/LACV3P**++ level the free energy of the carbene isomer is found to be 2.4 kcal mol^–1^ uphill from its alkene analogue. Interestingly the carbene CFCF_3_ ligand lies perpendicular to the (PNP)Rh plane, in contrast to the CF_2_ analogue described above, presumably due to steric interactions between the CF_3_ and the *cis*-PR_2_ groups.

**Fig. 8:**
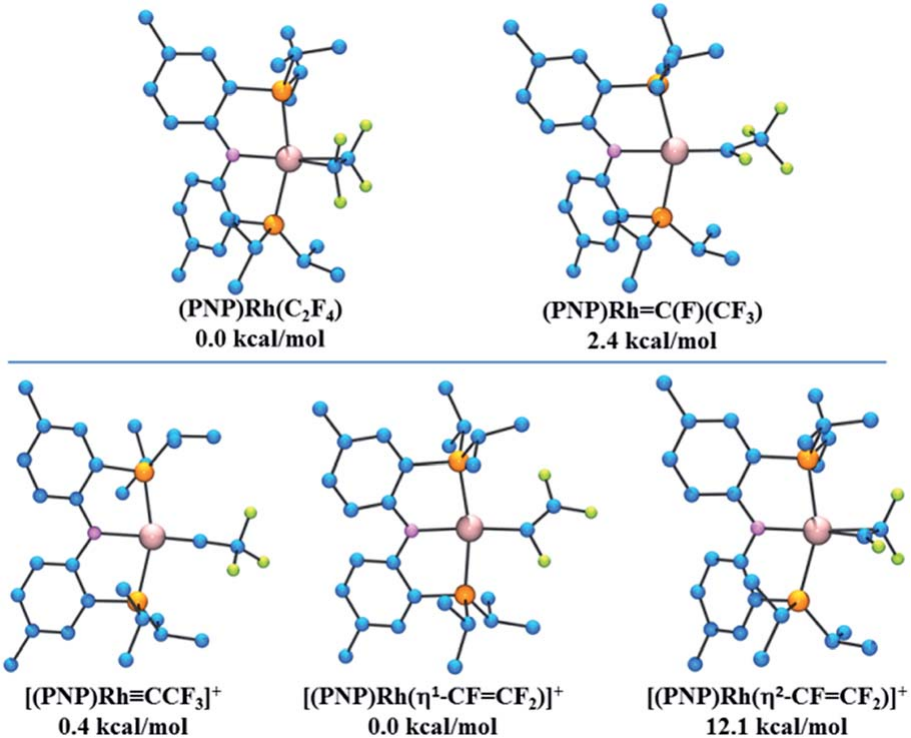
(Top) Calculated structures and relative energies for (PNP)Rh C_2_F_4_ isomers. (Bottom) Calculated structures and relative energies for proposed structures resulting from fluoride abstraction from (PNP)Rh=C(F)(CF_3_).

Potential products arising from fluoride abstraction from these isomeric complexes were also subjected to DFT evaluation. Abstraction of fluoride from (PNP)Rh(CFCF_3_) could occur from the α-position to yield a carbyne complex (PNP)Rh(CCF_3_)^+^, analogous to the characterized CF^+^ complex described above, or from the β-position to afford the corresponding isomeric η^1^-perfluorovinyl cation (PNP)Rh(CF=CF_2_)^+^. These are found to have almost identical free energies, with the carbyne complex lying only 0.4 kcal mol^–1^ higher than its perfluorovinyl isomer. An η^2^-perfluorovinyl isomer, the potential initial product of fluoride abstraction from the (PNP)Rh(C_2_F_4_) was found to lie 12.1 kcal mol^–1^ above its η^1^-perfluorovinyl analogue. Structures of all these compounds and their relative energies are provided in Fig. 8.

## Conclusions

In summary, we have shown that (PNP)Rh perfluorocarbene complexes can be synthesized by treating a Rh(i) precursor with Ruppert's reagent or a fluoroalkane containing a C–H bond. Using silylium reagents, a fluoride can be abstracted from (PNP)Rh=CF_2_ to form a cationic fluoromethylidyne. Thus the (PNP)Rh system conveniently allows synthesis and comparison of perfluoroolefin, perfluorocarbene, and perfluorocarbyne complexes. Using DFT calculations we were able to compare the natural localized molecular orbitals of these fluoroorganic complexes to their hypothetical hydrocarbon analogues, as well as to the CO and NO^+^ complexes. We established that the fluorine atoms on the carbene and carbyne ligands participate in π donation to the acceptor orbitals on carbon to compete with back donation from the metal. This resulted in a longer Rh–C bond in the fluorinated complexes compared to their hydrocarbon analogues. However, C_2_F_4_ was calculated to form a shorter Rh–C bond than the C_2_H_4_ complex. Calculated Wiberg bond indices also showed that although the unsaturated fluorocarbon ligands have bond orders greater than one to rhodium, the nitrogen *trans* to these ligands interacts with their antibonding orbital and decreases the bond order to less than a true double and triple bond.
