# Supplementary material for: Fluorocarbene, fluoroolefin, and fluorocarbyne complexes of Rh
Source: Chem Sci. 2017 Feb 16;8(4):3178–86. doi: 10.1039/c6sc05391b (PMC5414389; doi:10.1039/c6sc05391b)
Supplement: Supplementary file 1 [file SC-008-C6SC05391B-s001.pdf]

Electronic Supplementary Material (ESI) for Chemical Science.
This journal is © The Royal Society of Chemistry 2017

Electronic Supplementary Information

Fluorocarbene, Fluorocarbyne, and Fluorocarbyne Complexes of Rh

Christopher J. Pell[a], Yanjun Zhu[a], Rafael Huacuja[a], David E. Herbert[a], Russell P. Hughes[b], and Oleg V. Ozerov[a]**

[a]Department of Chemistry, Texas A&M University, College Station, Texas 77842

[b]Department of Chemistry, Dartmouth College, Hanover, New Hampshire 03755

Table of Contents

I. General Considerations S2

II. Synthesis and Characterization S3

III. X-ray Crystallography S26

IV. Computational Methods S28

V. Natural Localized Molecular Orbitals for (PNP)Rh=CH_2 S29

VI. Cartesian Coordinates S30

VII. References S58

I. General Considerations

Unless specified otherwise, all manipulations were performed under an argon atmosphere using standard Schlenk line or glove box techniques. Toluene, pentane, and isooctane were dried and deoxygenated (by purging) using a solvent purification system by MBraun and stored over molecular sieves in an Ar-filled glove box. C_6D_6 and $CDCl_3$ was dried over and distilled from Na/K/Ph_2CO/18-crown-6 and stored over molecular sieves in an Ar-filled glove box. Fluorobenzene and benzotrifluoride were dried with and then distilled from CaH_2 and stored over molecular sieves in an Ar-filled glove box. Me_3SiCF_3 was degassed prior to use and stored in an Ar-filled glove box. CsF was grounded and dried at 130 °C under vacuum for 2 days. (PNP)Rh(TBE)[1], $[(Et_3Si)_2(SO_3CF_3)][HCB_{11}Cl_{11}]$[2], and $[Ph_3C][HCB_{11}Cl_{11}]$[2], was prepared according to the published procedures. $[(PNP)Rh]_2(\mu\text{-}N_2)$ was previously characterized.[3] All other chemicals were used as received from commercial vendors unless otherwise specified.

<u>III. Synthesis and Characterization</u>

Synthesis of (PNP)Rh=CF_2. <u>Method 1</u>. (PNP)Rh(TBE) (105 mg, 0.170 mmol), Me_3SiCF_3 (50.0 μL, 0.340 mmol) and CsF (25.0 mg, 0.170 mmol) were placed in a Teflon flask in about 3 mL C_6D_6. The reaction mixture was stirred vigorously and heated at 70 °C for 6 d. NMR analysis revealed the presence of 85% of (PNP)Rh=CF_2 and 14% of (PNP)Rh(C_2F_4) together with 1% of (PNP)Rh(CO). The same distribution of products (85% (PNP)Rh=CF_2, 14% (PNP)Rh(C_2F_4), and 1% (PNP)Rh(CO)) was obtained when the mixture was heated for 3 d at 80 °C. The reaction mixture was passed through Celite. The volatiles were removed under vacuum and the residue was extracted with pentane. The pentane solution was passed through Celite again. The volatiles were removed under vacuum. The residue was dissolved in isooctane and placed in the freezer at -35 ^{o}C. Small amount of precipitate was formed the next day. The supernatant was filtered through Celite, then the volatiles were removed and the residue dried under vacuum. NMR analysis indicated > 98% purity of (PNP)Rh=CF_2 (51 mg, 52% yield). <u>Method 2</u>. (PNP)Rh(TBE) (400 mg, 0.65 mmol) was dissolved in toluene in a 50 mL PTFE screw-top flask with a PTFE-coated stir bar. The solution was degassed and filled with 1 atm of CHF_3 and heated overnight at 80 °C. The volatiles were removed under vacuum in the morning and the product was extracted with pentane and filtered through a pad of silica and Celite. The volatiles were removed to yield a dark red powder (226 mg) that was composed of 3% (PNP)Rh(CO), 81% (PNP)Rh(=CF_2), and 16% [(PNP)Rh]$_2$(μ-N_2). ^{1}H NMR (C_6D_6): δ 7.66 (d, J = 8 Hz, 2H, (PNP)Aryl-H), 7.10 (br, 2H, (PNP)Aryl-H), 6.80 (d, J = 8 Hz, 2H, (PNP)Aryl-H), 2.43 (m, 4H, $CH(CH_3)_2$), 2.19 (s, 6H, Ar-CH3), 1.27 (dvt (app. q.), J = 8 Hz, 12 H, $CH(CH_3)_2$), 1.13 (dvt (app. q.), J = 8 Hz, 12 H, $CH(CH_3)_2$). ^{31}P{^{1}H}NMR (C_6D_6) : 53.7 (dt, $J_{Rh\text{-}P}$ = 144 Hz, $J_{F\text{-}P}$ = 30 Hz). ^{19}F NMR (C_6D_6): 95.6 (dt, $J_{Rh\text{-}F}$ = 49 Hz, $J_{P\text{-}F}$ = 30 Hz). ^{13}C{^{1}H}NMR (C_6D_6):

206.3 (dtt, $J_{Rh\text{-}C}$ = 85 Hz, $J_{C\text{-}P}$ = 12 Hz, $J_{C\text{-}F}$ = 454 Hz), 162.3 (t, J = 11 Hz), 132.4, 131.9, 125.8 (t, J = 3 Hz), 122.3 (t, J = 18 Hz), 116.1 (t, J = 3 Hz), 25.4 (t, J = 11 Hz), 20.5, 19.3 (t, J = 3 Hz), 18.5. Elem. Anal. Found (Calculated) for $C_{27}H_{40}F_2NP_2Rh$: C, 55.81 (55.77); H, 6.96 (6.93); N, 2.35 (2.41).

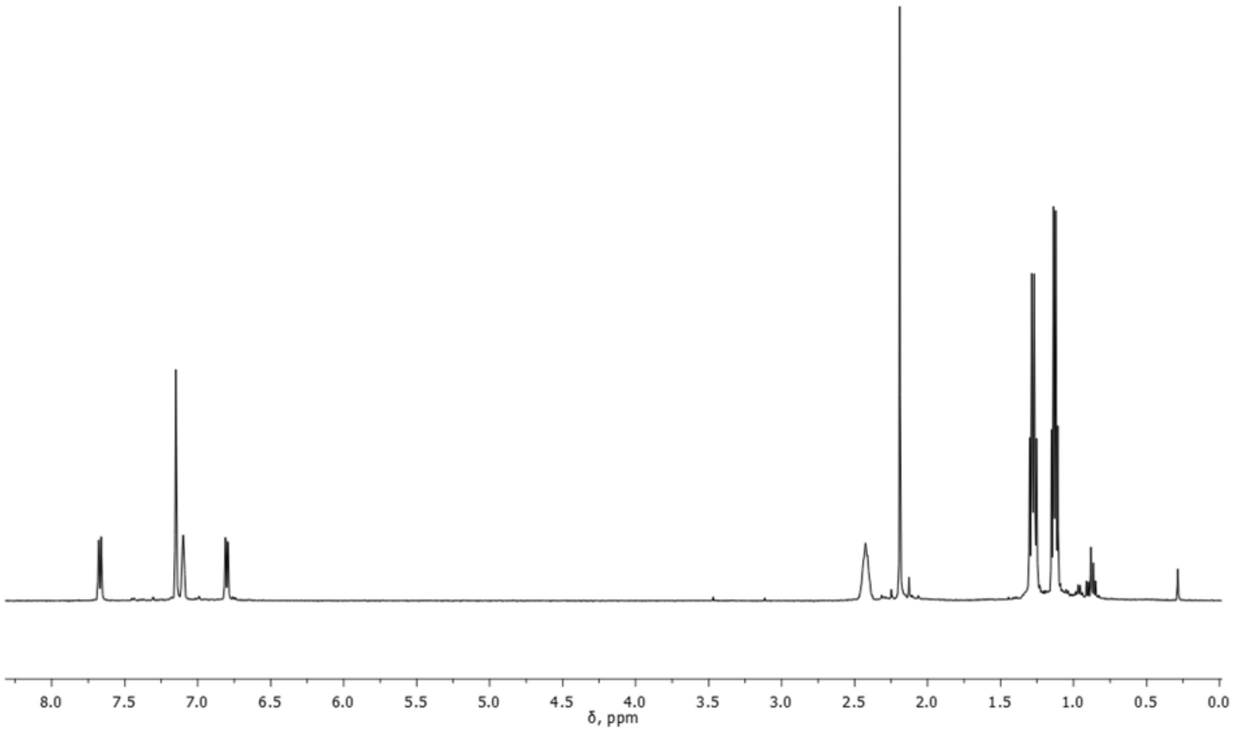

Figure S1: ^{1}H NMR spectrum of (PNP)Rh=CF_2 in C_6D_6. Residual pentane is visible at 0.86 ppm. Residual isooctane is visible at 0.88 ppm, 0.90 ppm, 0.92 ppm. Residual silicone grease is visible at 0.28 ppm.

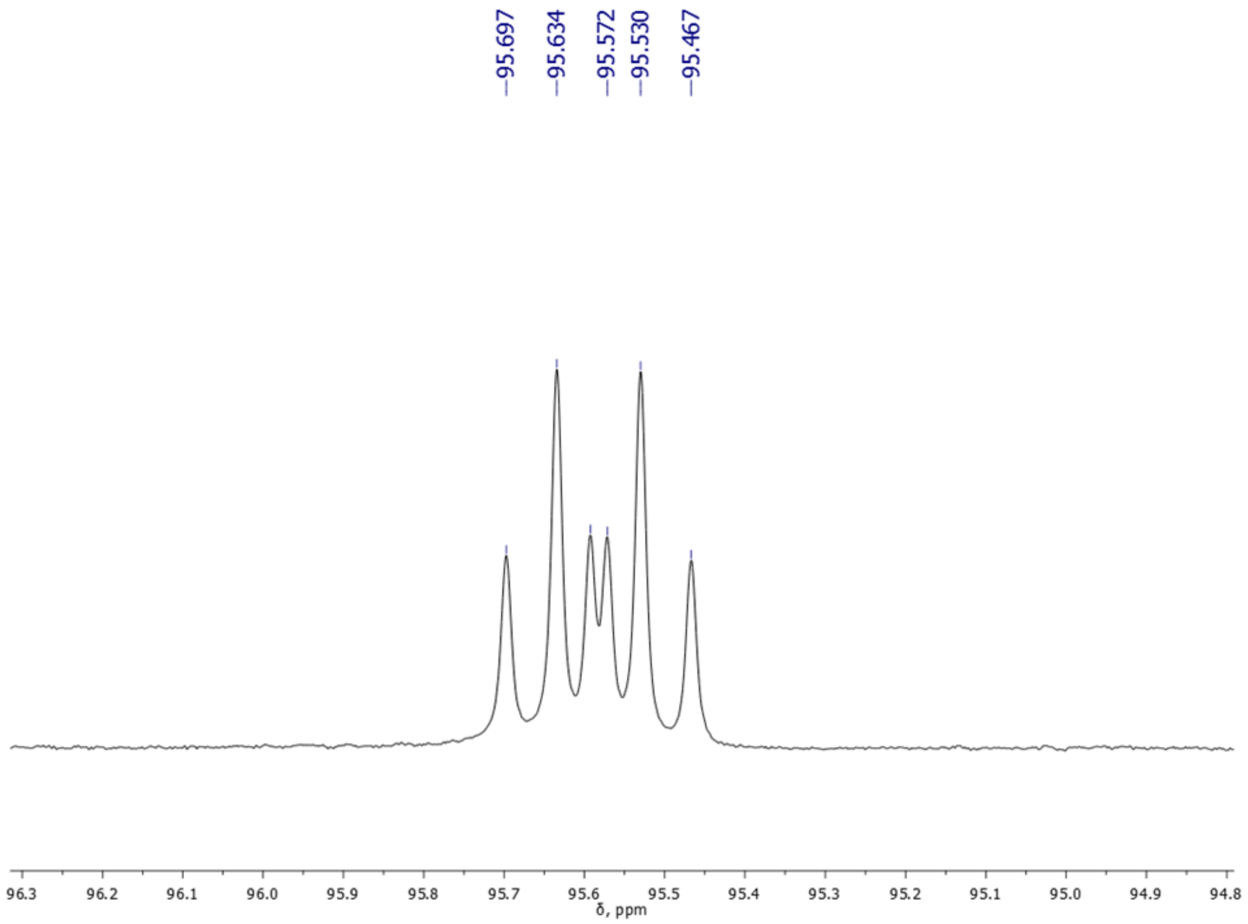

Figure S2: ^{19}F NMR spectrum for (PNP)Rh=CF_2 in C_6D_6.

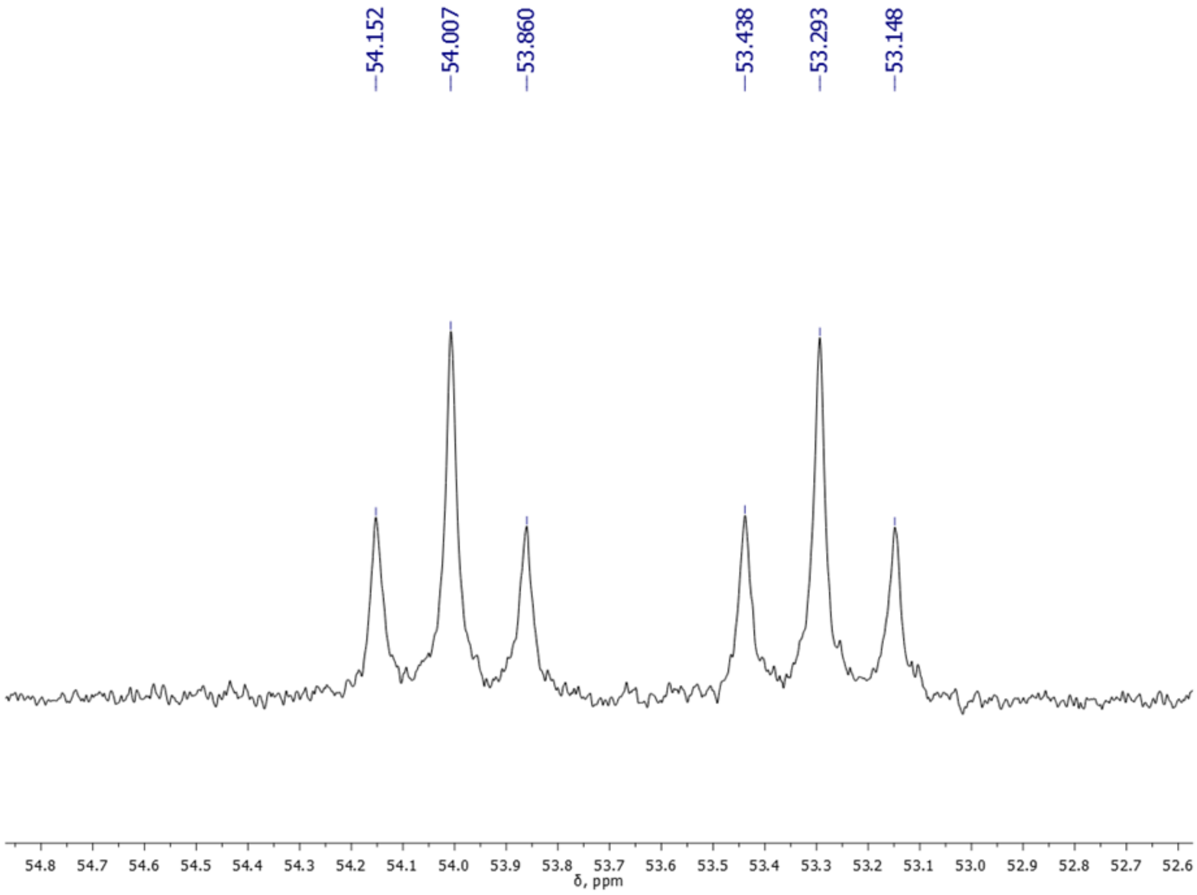

Figure S3: $^{31}P\{^{1}H\}$ NMR spectrum of (PNP)Rh=CF_2 in C_6D_6.

Attempted Hydrolysis of (PNP)Rh=CF_2. A 20 mg mixture [91% (PNP)Rh(=CF_2), 5% (PNP)Rh(CO), 4% (PNP)Rh]$_2$(μ-N_2) was dissolved in C_6D_6 and H_2O (10 μL) was added. The solution was left for 24 h at room temperature. No decomposition of (PNP)Rh(=CF_2) to (PNP)Rh(CO) was observed by $^{31}P\{^{1}H\}$ NMR spectroscopy. Heating the sample overnight at 80 °C also led to no visible decomposition of (PNP)Rh(=CF_2) to (PNP)Rh(CO), with (PNP)Rh(=CF_2) still accounting for >90% of the reaction mixture by $^{31}P\{^{1}H\}$ NMR spectroscopy.

Treatment of (PNP)Rh(CO) with Me_3SiCF_3 and CsF. (PNP)Rh(CO) (28 mg, 0.050 mmol) was dissolved in toluene in a PTFE screw-top vial and treated with Me_3SiCF_3 (30 μL,

0.20 mol) and CsF (16 mg, 0.10 mmol) and heated at 80 °C for 3 days. No reaction was observed to take place.

Treatment of (PNP)Rh=CF_2 with Me_3SiCF_3 and CsF. A 1:1 mixture of (PNP)Rh=CF_2 and (PNP)Rh(CO) (28 mg, 0.050 [Rh] (0.025 mmol per species)) was dissolved in toluene in a PTFE screw-top vial and treated with Me_3SiCF_3 (30 μL, 0.20 mol) and CsF (16 mg, 0.10 mmol) and heated at 80 °C for 3 days. Analysis by $^{31}P\{^1H\}$ NMR spectroscopy using (PNP)Rh(CO) as an internal integration standard showed that no reaction had occurred.

Treatment of 1,2-dibromo-tetrafluoroethane with Zinc. Zinc (10 mg, 0.15 mmol) was added to a J. Young tube with 600 μL of THF. 1,2-dibromo-tetrafluoroethane (12 μL, 0.10 mmol) was added to the solution. Bubbles could be seen indicating the formation of tetrafluoroethylene upon mixing the heterogeneous mixture. ^{19}F NMR spectroscopy was used to observe tetrafluoroethylene as a singlet resonating at -133.67 ppm in THF.

Observation of tetrafluoroethylene in C_6D_6. Zinc (10 mg, 0.15 mmol) was added to a J. Young tube with 600 μL of THF. 1,2-dibromo-tetrafluoroethane (12 μL, 0.10 mmol) was added to the solution. The solution was then frozen in liquid nitrogen and the headspace evacuated under vacuum. The solution was thawed and shaken. Bubbles could be seen forming around the solid zinc particles. After 20 minutes, the produced tetrafluoroethylene was vacuum transferred to another J. Young tube containing 600 μL of C_6D_6. ^{19}F NMR spectroscopy was used to observe tetrafluoroethylene as a singlet resonating at -132.48 ppm in C_6D_6.

Treatment of Me_3SiCF_3 with CsF in C_6D_6. CsF (25 mg, 0.14 mmol) was added to a J. Young tube containing C_6D_6. Me_3SiCF_3 (50 μL, 0.39 mmol) was added to the J. Young tube and the solution was heated at 80 °C for 1 h. ^{19}F NMR spectroscopy showed the formation of

tetrafluoroethylene. The reaction was heated at 80 °C for 3 d. ^{19}F NMR spectroscopy showed that tetrafluoroethylene (δ = -132.63 ppm) and Me_3SiF (δ = -158.00) were the two major products.

Synthesis of (PNP)Rh(CF_2=CF_2). Method 1. (PNP)Rh(TBE) (300 mg, 0.49 mmol) was dissolved in THF with powdered zinc (240 mg, 3.67 mmol) in a Schlenk flask. The mixture is heated to 50 °C and a solution of 1,2-dibromo-tetrafluoroethane (290 μL, 2.42 mmol) is added dropwise. The solution immediately starts to bubble with the formation of tetrafluoroethylene. The reaction was heated overnight and the volatiles were removed under vacuum in the morning. The product was extracted with toluene and filtered through a plug of silica and Celite. The volatiles were removed again and the product was extracted with pentane and filtered through Celite. The volatiles were removed and the product was isolated as a light orange solid (138 mg, 45% yield). Method 2. An alternative synthesis was performed by dissolving (PNP)Rh(TBE) (300 mg, 0.49 mmol) in THF in a PTFE screw-cap flask. Powdered zinc (240 mg, 3.67 mmol) was added to another flask in THF, and 1,2-dibromo-tetrafluoroethane (290 μL, 2.42 mmol) was added dropwise. After 30 minutes of stirring the 1,2-dibromo-tetrafluoroethane over zinc at 50 °C, the produced tetrafluoroethylene was vacuum transferred to the first flask containing (PNP)Rh(TBE). The reaction was stirred overnight at 50 °C and the volatiles were removed under vacuum in the morning. The product was filtered through silica and Celite in THF and washed with pentane to yield the product as a light orange solid (197 mg, 64% yield). ^1H NMR (C_6D_6): δ 7.45 (d, J = 8.5 Hz, 2H, Ar-*H*), 6.81 (br, 2H, Ar-*H*), 6.75 (d, J = 8.5 Hz, 2H, Ar-*H*), 2.30 (br, 4H, PC*H*Me_2), 2.13 (s, 6H, Ar-*Me*), 1.30 (br, 12H, PCH*Me*$_2$), 0.96 (overlapping dt (app. q), J = 7 Hz, 12H, PCH*Me*$_2$). $^{31}P\{^1H\}$ NMR (C_6D_6): 52.3 (dqui., $J_{Rh\text{-}P}$ = 137 Hz, $J_{F\text{-}P}$ = 23 Hz); ^{19}F NMR (C_6D_6): 100.7 (br); $^{13}C\{^1H\}$ NMR ($CDCl_3$): 161.5 (dvt, $J_{C\text{-}P}$ = 11 Hz, $J_{C\text{-}Rh}$ = 2 Hz, *C*-

N), 132.3 (s, *Ar*), 131.8 (s, *Ar*), 126.7 (vt, J_{C-P} = 3 Hz, *Ar*), 119.2 (vt, J_{C-P} = 20 Hz, *Ar*), 115.1 (vt, J_{C-P} = 5 Hz, *Ar*), 111.37 (m, C_2F_4), 23.8 (br, P*C*HMe$_2$), 20.6 (s, Ar-*Me*), 18.5 (br, P*C*HMe$_2$), 16.7 (s, PCH*Me*$_2$). Elem. Anal. Found (Calculated) for $C_{28}H_{40}F_4NP_2Rh$: C, 53.39 (53.26); H, 6.47 (6.38).

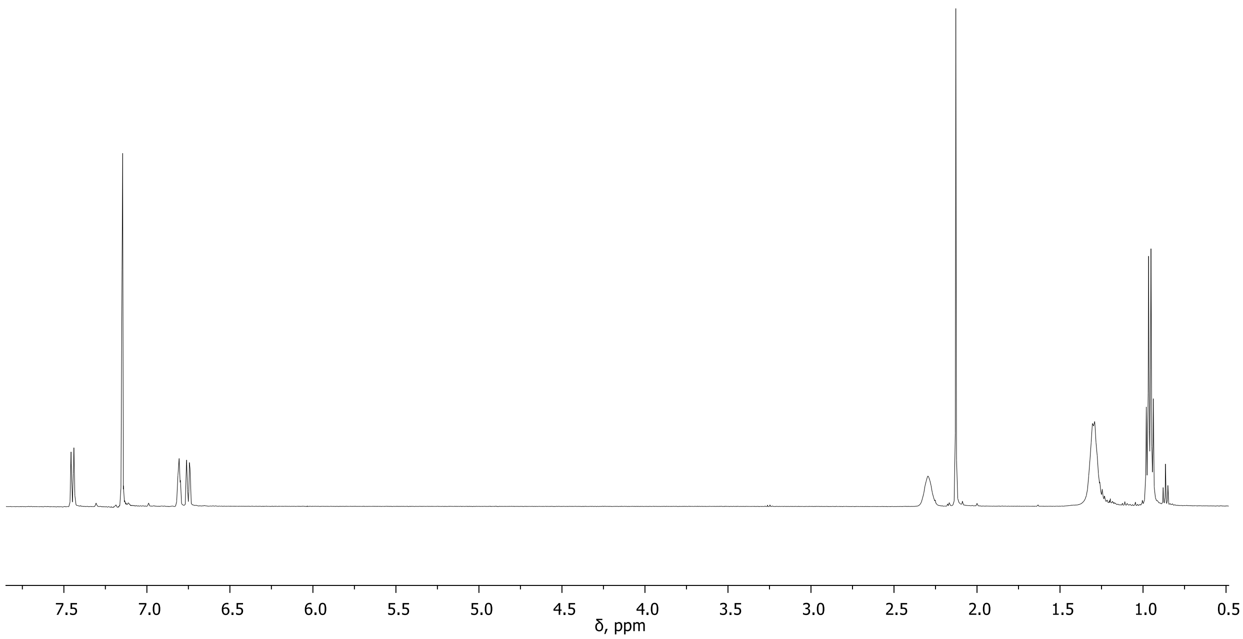

Figure S4: ^{1}H NMR of (PNP)Rh(CF_2=CF_2) in C_6D_6. Pentane resonances visible at 0.87 and 1.23 ppm.

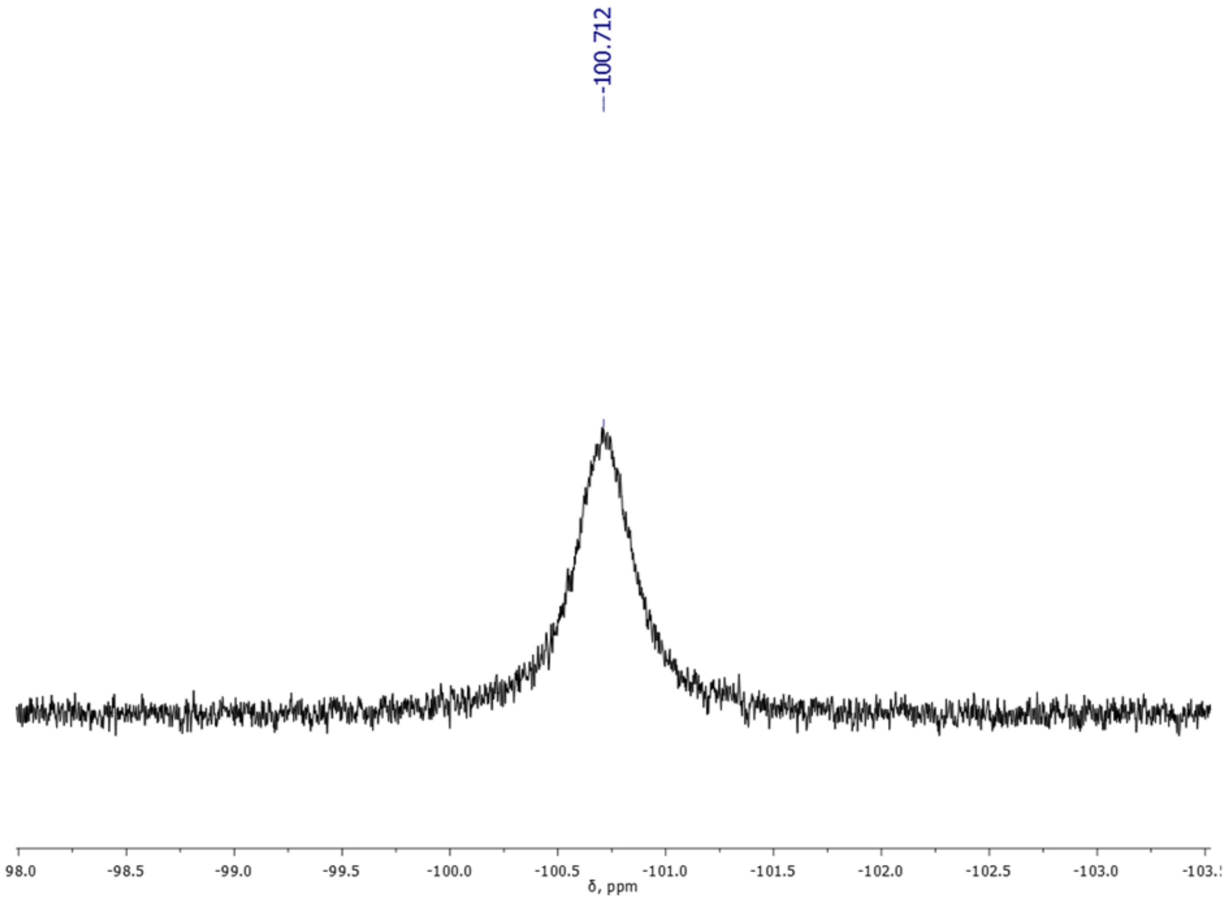

Figure S5: ^{19}F NMR spectrum of (PNP)Rh(CF_2=CF_2) in C_6D_6.

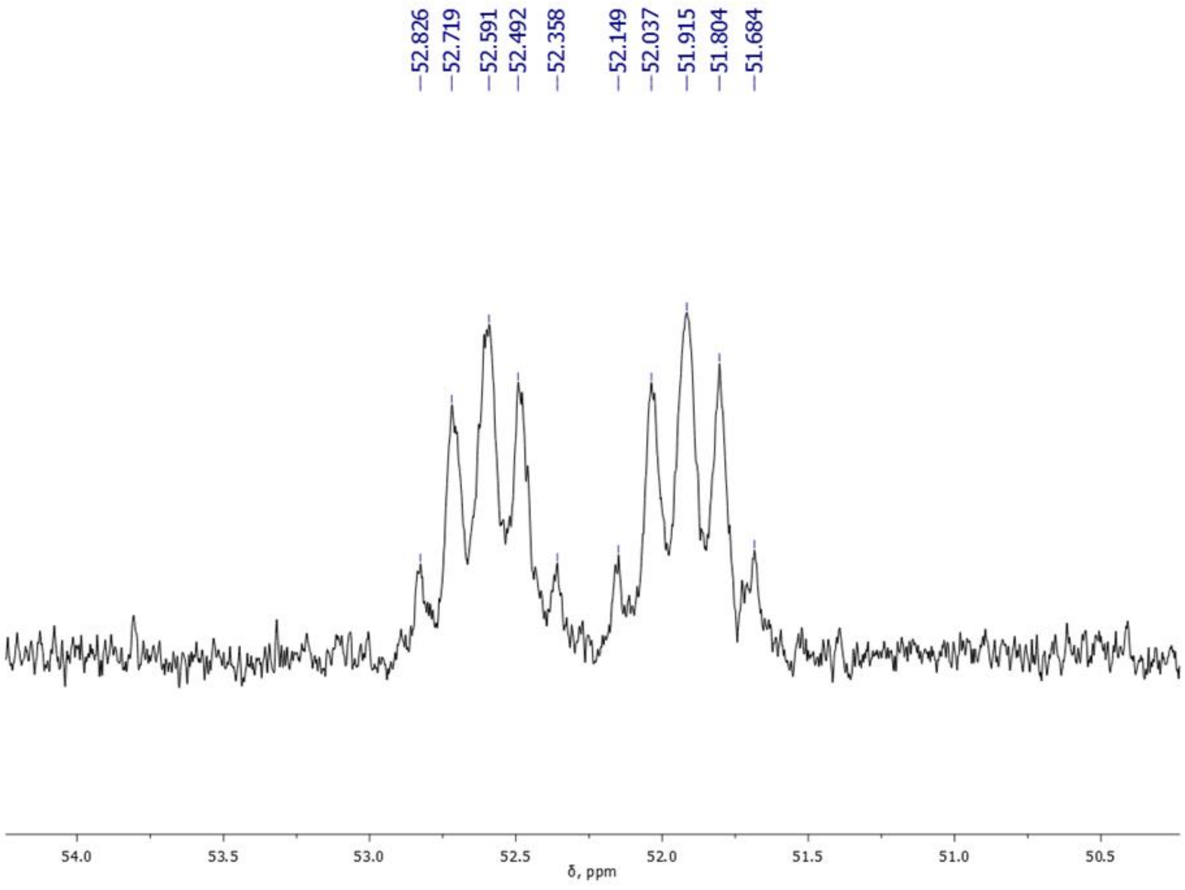

Figure S6: $^{31}P\{^1H\}$ NMR spectrum of (PNP)Rh(CF_2=CF_2) in C_6D_6.

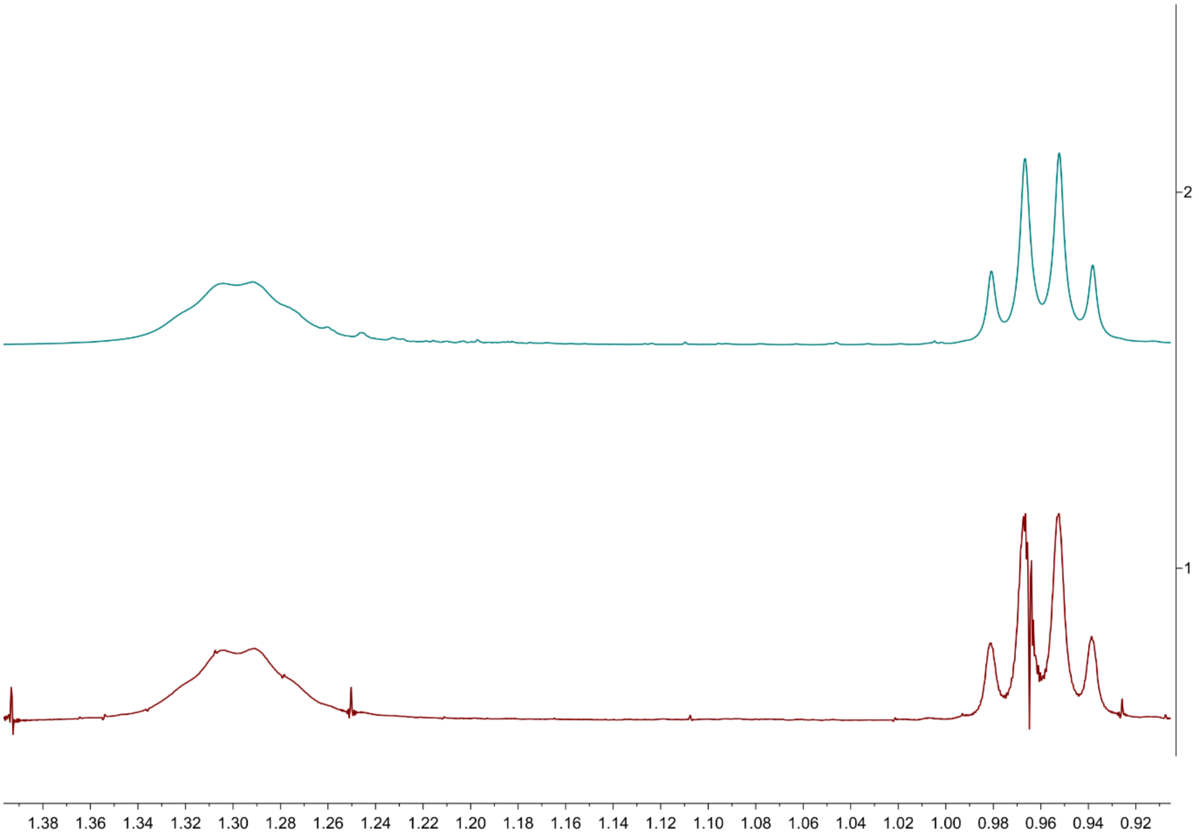

Figure S7: $^{1}H\{^{19}F\}$ NMR spectrum of the isopropyl methyl signals of (PNP)Rh(CF_2=CF_2) in C_6D_6 (bottom). ^{1}H NMR spectrum of the isopropyl methyl signals of (PNP)Rh(CF_2=CF_2 in C_6D_6).

Treatment of (PNP)Rh(TBE) with C_2HF_5 in a J. Young tube. (PNP)Rh(TBE) (22 mg, 0.036 mmol) was dissolved in C_6D_6 and the solution was degassed and charged with 1 atm of C_2H F_5. The solution was heated at 80 °C overnight. Analysis by $^{31}P\{^{1}H\}$ NMR spectroscopy in the morning showed 52% conversion to [(PNP)Rh]$_2$(μ-N_2) and (PNP)Rh(N_2), 9% (PNP)Rh(CO), 27% (PNP)Rh=C(F)(CF_3).

Synthesis of (PNP)Rh(=C(F)(CF_3)). (PNP)Rh(TBE) (200 mg, 0.32 mmol) was dissolved in benzotrifluoride (5 mL) in a 25 mL PTFE screw top flask, and the solution was degassed under vacuum. A 25 mL PTFE screw top flask was filled with 1 atm of C_2HF_5 and

frozen in liquid nitrogen, once the C_2HF_5 (melting point = -103 °C) was frozen in the flask, the volatiles were removed under vacuum to remove dinitrogen from the gas mixture. The C_2HF_5 was freeze-pumped-thawed two more times and then vacuum transferred to the flask containing the (PNP)Rh(TBE) solution. The solution was then heated overnight at 80 °C. The flask was cooled to room temperature and the volatiles were removed under vacuum. A portion of the resulting greenish blue solid was dissolved in C_6D_6 and analyzed by $^{31}P\{^1H\}$ NMR, which displayed a mixture composed of 62% (PNP)Rh=C(F)(CF_3), 1% (PNP)Rh(CO), and 37% (PNP)Rh(TBE). The solid was redissolved in benzotrifluoride and treated with C_2HF_5 following the previously described procedure. The reaction was heated overnight at 80 °C, and the volatiles were removed under vacuum in the morning. The resulting dark blue solid was dissolved in pentane and filtered through a pad of silica and Celite. The volatiles were removed to yield a blue powder (156 mg, 78% yield >90% pure). Analysis by $^{31}P\{^1H\}$ NMR spectroscopy determined the mixture to be >90% (PNP)Rh=C(F)(CF_3), the rest of the mixture was composed of (PNP)Rh(CO). Dissolving the mixture in pentane and placing in a -35 °C freezer overnight formed clover-shaped crystals. Analysis of the crystals by $^{31}P\{^1H\}$ NMR spectroscopy showed the same distribution of products (>90% (PNP)Rh=C(F)(CF_3) and <10% (PNP)Rh(CO)). Method 2. (PNP)Rh(TBE) (200 mg, 0.32 mmol) was dissolved in benzotrifluoride (5 mL) in a 25 mL PTFE screw top flask, and the solution was degassed under vacuum. A 50 mL PTFE screw top flask was filled with 1 atm of C_2HF_5 and frozen in liquid nitrogen, once the C_2HF_5 (melting point = -103 °C) was frozen in the flask, the volatiles were removed under vacuum to remove dinitrogen from the gas mixture. The C_2HF_5 was freeze-pumped-thawed two more times and then vacuum transferred to a 25 mL PTFE capped flask containing the (PNP)Rh(TBE) solution. The solution was then heated for 24 h at 80 °C. The flask was cooled to room temperature and an

aliquot of the reaction mixture was transferred to J. Young tube for NMR analysis. $^{31}P\{^1H\}$ NMR spectroscopy showed a mixture containing 25% (PNP)Rh(CO), 52% (PNP)Rh=C(F)(CF_3), and 13% (PNP)Rh=CF_2. ^1H NMR (C_6D_6): 7.57 (dvt, *J* = 8.5 Hz, J_{H-P} = 2 Hz, 2H, Ar-*H*), 7.05 (m, 2H, Ar-*H*), 6.77 (dd, *J* = 9 Hz, *J* = 1.5 Hz, 2H, Ar-*H*), 2.53 (m, 4H, PC*H*Me$_2$), 2.18 (s, 6H, Ar-*Me*), 1.28 (overlapping dvt (app. q), *J* = 7.5 Hz, PCH*Me*$_2$), 1.05 (overlapping dvt (app. q), *J* = 7 Hz, PCH*Me*$_2$); ^{19}F NMR (C_6D_6): 75.6 (br. t, *J* = 39.5 Hz, 1F, Rh=C(*F*)(CF_3)), -74.2 (d, *J* = 11.3 Hz, 3F, Rh=C(F)(C*F*$_3$)); $^{31}P\{^1H\}$ NMR (C_6D_6): 55.4 (dd, J_{Rh-P} = 149.3 Hz, J_{F-P} = 41.0 Hz); $^{13}C\{^1H\}$ NMR (C_6D_6): 225.0 (m, Rh=*C*), 161.3 (dvt, J_{C-P} = 12 Hz, J_{C-Rh} = 2 Hz, *C*-N), 132.3 (s, *Ar*-H), 132.1 (s, *Ar*-H), 126.2 (vt, J_{C-P} = 3 Hz, *Ar*), 121.6 (vt, J_{C-P} = 19 Hz, *Ar*), 116.8 (vt, J_{C-P} = 5 Hz, *Ar*), 25.3 (vt, J_{C-P} = 11 Hz, P*C*HMe$_2$), 20.5 (s, Ar-*Me*), 19.4 (vt, J_{C-P} = 3 Hz, P*C*HMe$_2$), 18.3 (s, PCH*Me*$_2$).

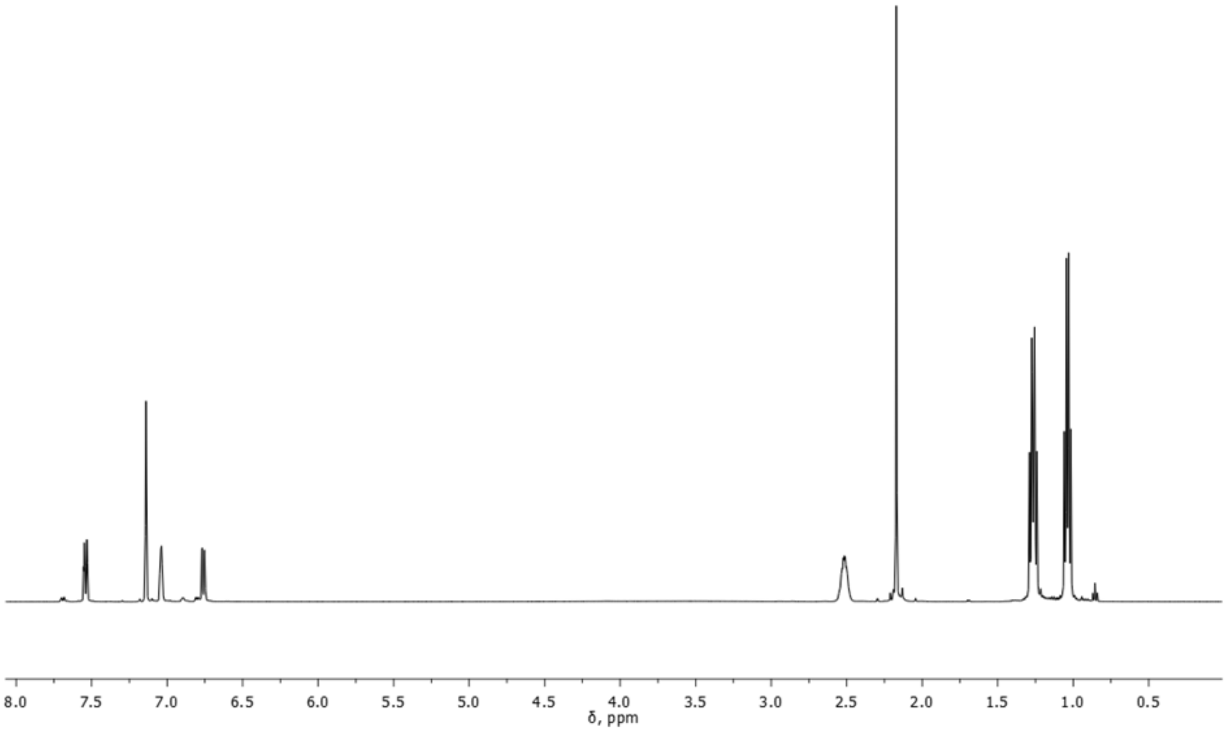

Figure S8: ^{1}H NMR of (PNP)Rh=C(F)(CF_3) in C_6D_6 with about 7% (PNP)Rh(CO). Pentane resonances are also visible at 0.87 and 1.23 ppm.

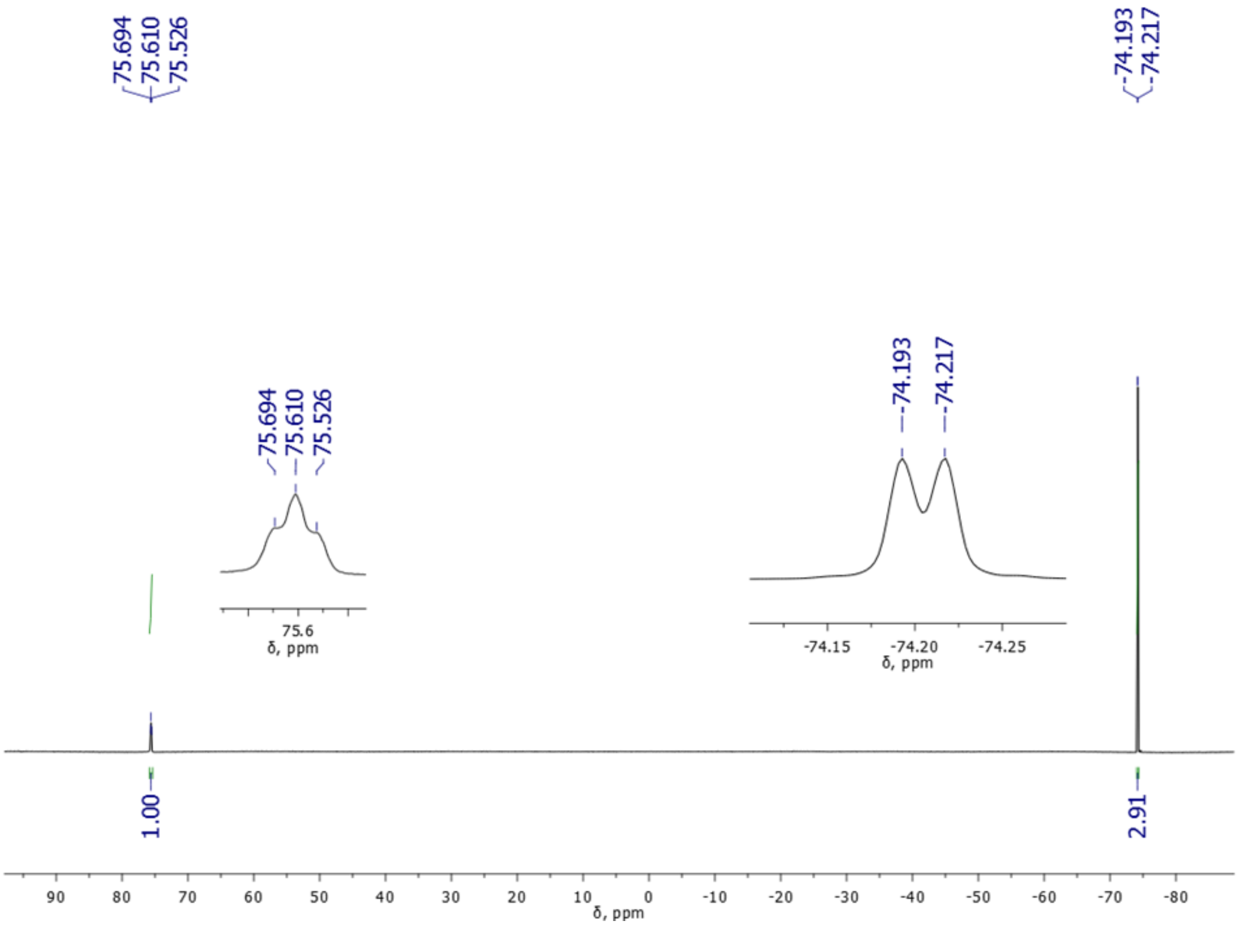

Figure S9: ^{19}F NMR spectrum of (PNP)Rh=C(F)(CF_3) in C_6D_6.

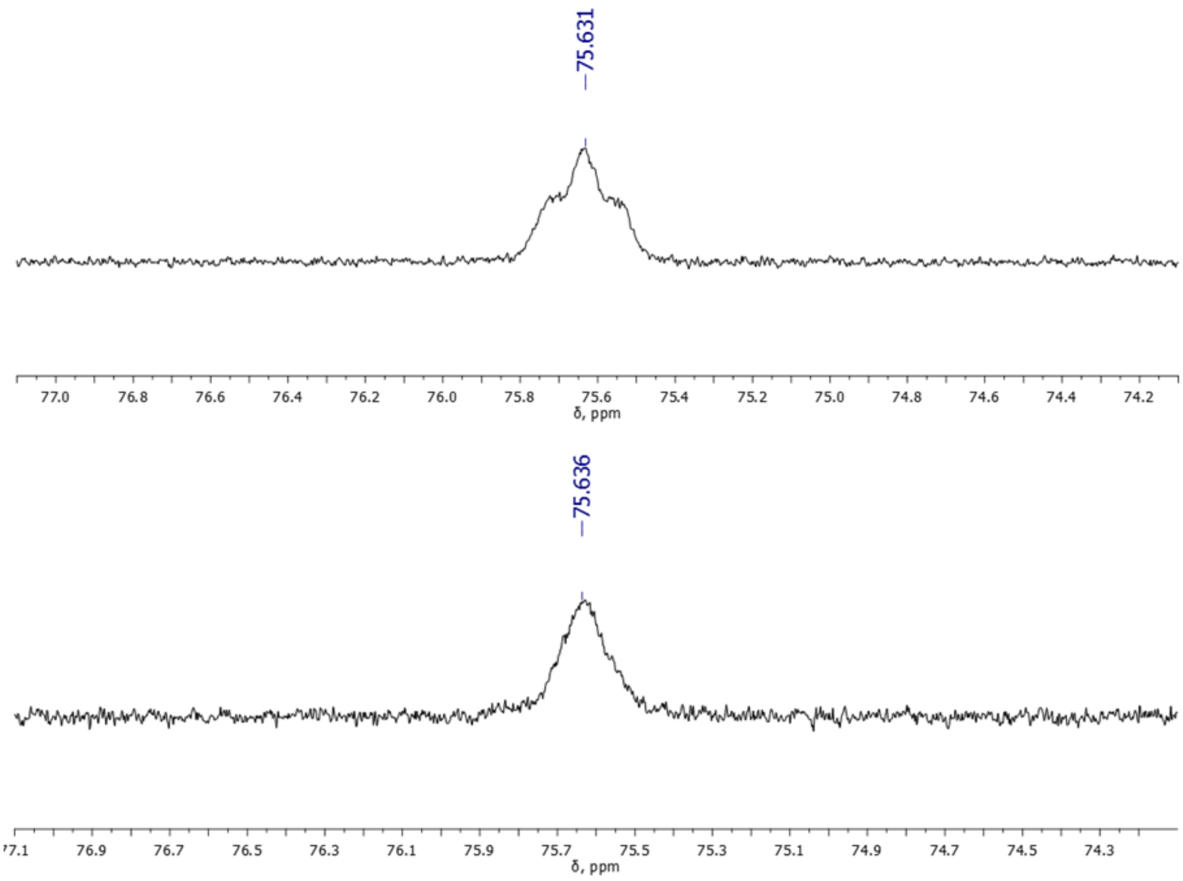

Figure S10: $^{19}F\{^{31}P\}$ NMR spectrum of (PNP)Rh=C(F)(CF_3) in C_6D_6 (below) ^{19}F NMR spectrum of (PNP)Rh=C(F)(CF_3) in C_6D_6.

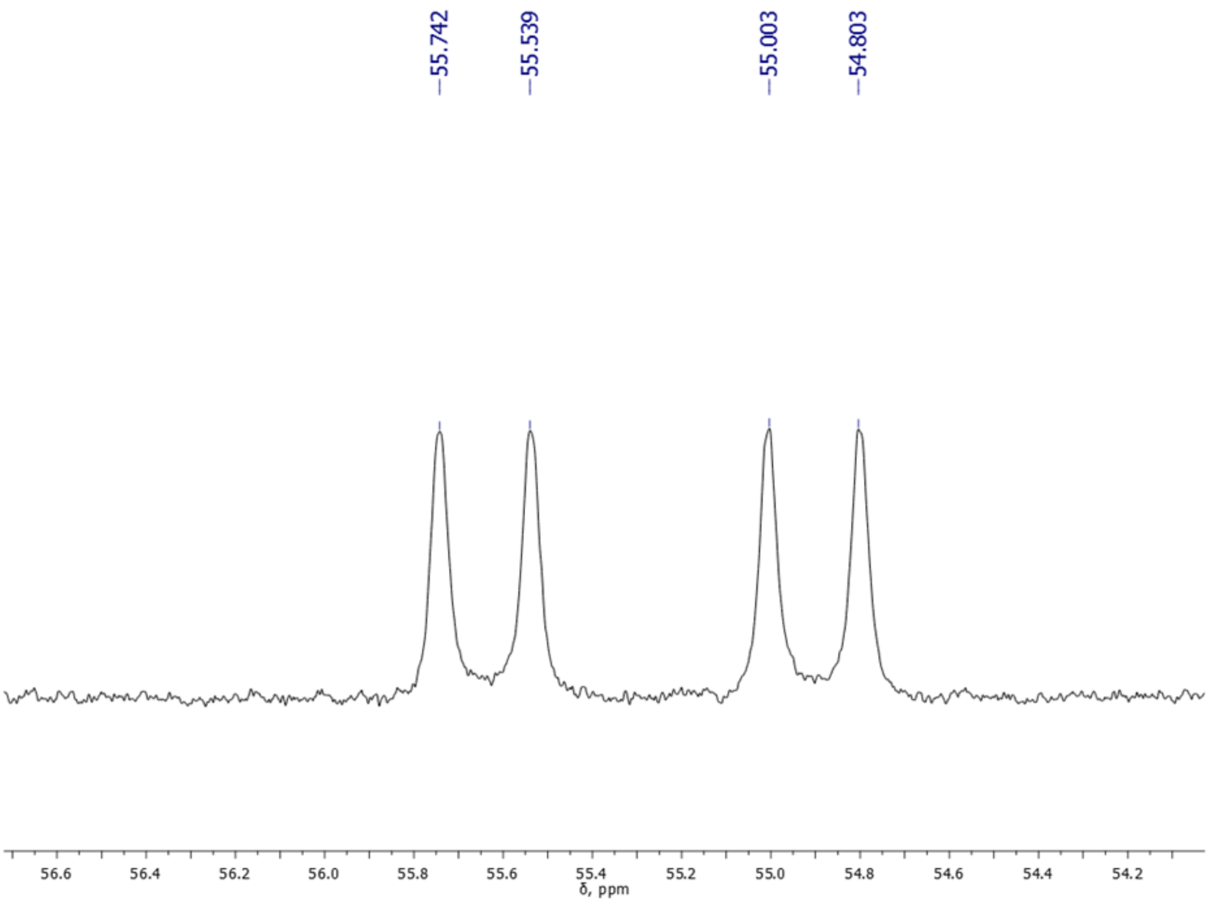

Figure S11: $^{31}P\{^{1}H\}$ NMR spectrum of (PNP)Rh=CF(CF_3) in C_6D_6.

Synthesis of [(PNP)Rh≡CF][$CHB_{11}Cl_{11}$]. [Ph_3C][$CHB_{11}Cl_{11}$] (31.6 mg, 0.041 mmol) was dissolved in about 1 mL of PhF in a flask followed by the addition of Et_3SiH (8.0 μL, 0.049 mmol). The yellow solution changed to colorless within minutes. (PNP)Rh(=CF_2) (24 mg, 0.041 mmol) was added to the reaction mixture. The solution changed to green/blue immediately. The bluish solid was precipitated out by adding excess pentane to the solution. The supernatant was decanted. The solid was washed with pentane (3x3 mL) and dried under vacuum. Yield, 27 mg, 61%. The X-ray quality crystals were obtained by carefully laying pentane to 1,2-difluorobenzene solution of the title compound at room temperature. ^{1}H NMR (C_6D_6/1,2-difluorobenzene, 1,2-difluorobenzene was added to increase the solubility and it overlapped with

the aromatic resonances of the title compound. Only aliphatic region was reported): δ 2.66 (br, 1H, C*H*$B_{11}Cl_{11}$), 2.41 (m, 4H, C*H*Me_2), 2.18 (s, 6H, Ar-C*H_3*), 1.03 (app. q, *J* = 11 Hz, 12H, CH*Me_2*), 0.90 (app. q, *J* = 11 Hz, 12H, CH*Me_2*). ^{31}P{^{1}H} NMR (C_6D_6/1,2-difluorobenzene): δ 89.3 (dd, $J_{Rh\text{-}P}$ = 106 Hz, $J_{F\text{-}P}$ = 14 Hz). ^{19}F NMR: δ 66.2 (dt, $J_{Rh\text{-}F}$ = 136 Hz, $J_{P\text{-}F}$ = 14 Hz). ^{13}C{^{1}H,^{31}P} NMR (C_6D_5Br/1,2-difluorobenzene): δ 160.1(s), 134.1(s), 132.5 (s), 116.2 (s) 47.3 (broad s, C-H) 26.3 (s, *C*HMe_2), 20.0(s, Ar-CH_3), 18.7 (s, CH*Me_2*), 17.6 (s, CH*Me_2*). (2 aromatic signals are overlaping with solvent). Elem. Anal. Found (Calculated) for $C_{28}H_{41}B_{11}Cl_{11}FNP_2Rh$: C, 31.02 (31.01); H, 3.80 (3.81).

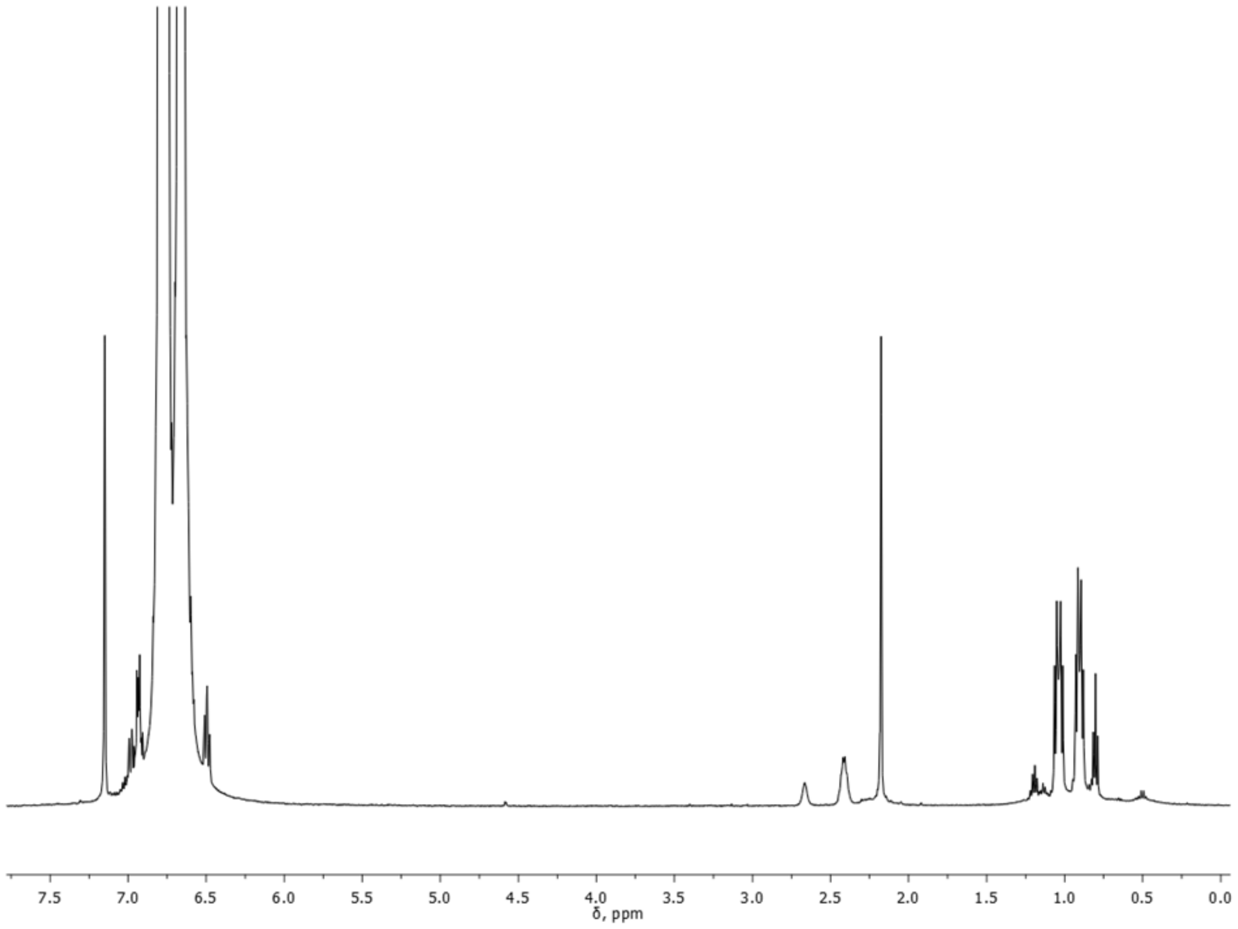

Figure S12: ^{1}H NMR spectrum of [(PNP)Rh≡CF][$CHB_{11}Cl_{11}$] in C_6D_6/1,2-difluorobenzene. Pentane resonances are visible at 0.79 and 1.19 ppm.

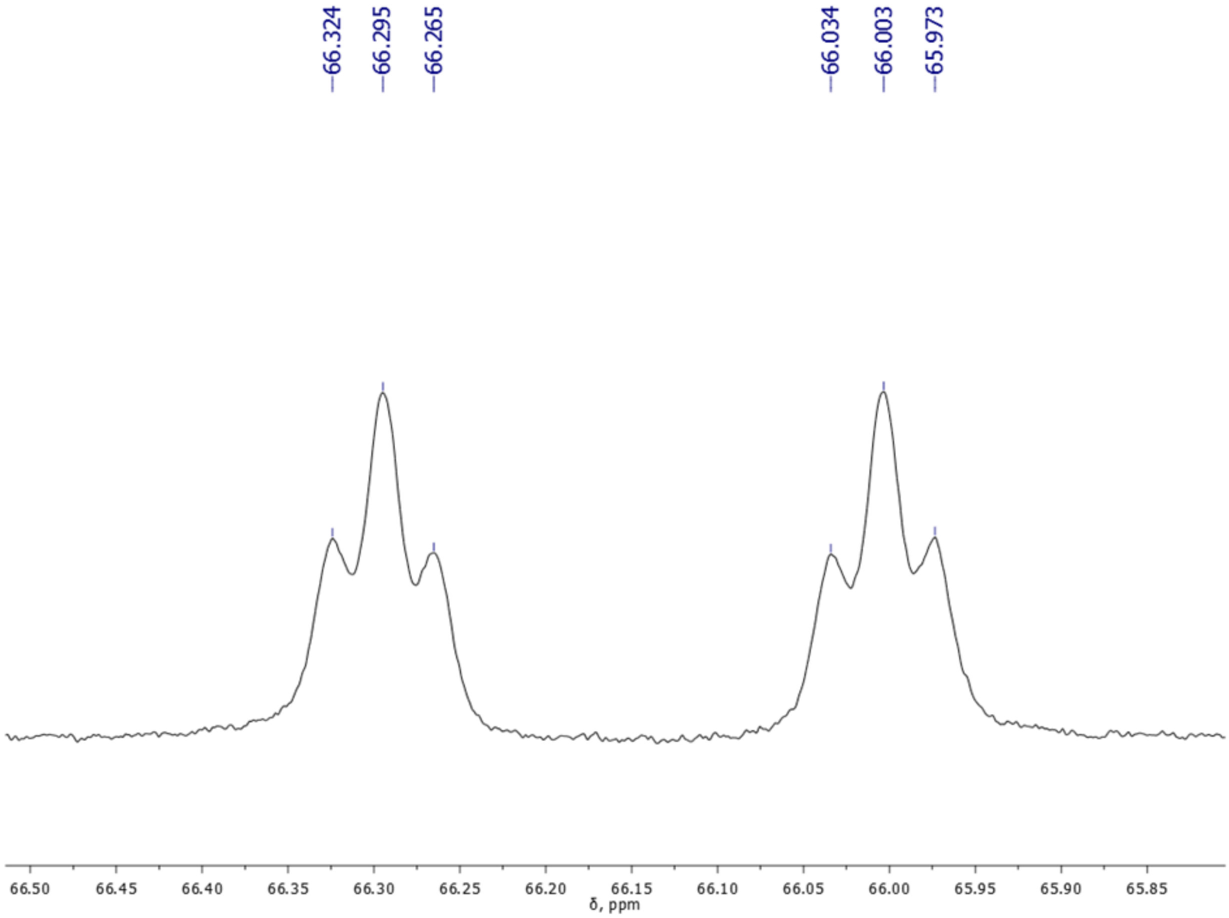

Figure S13: ^{19}F NMR spectrum of [(PNP)Rh≡CF][$CHB_{11}Cl_{11}$] in C_6D_6/1,2-difluorobenzene.

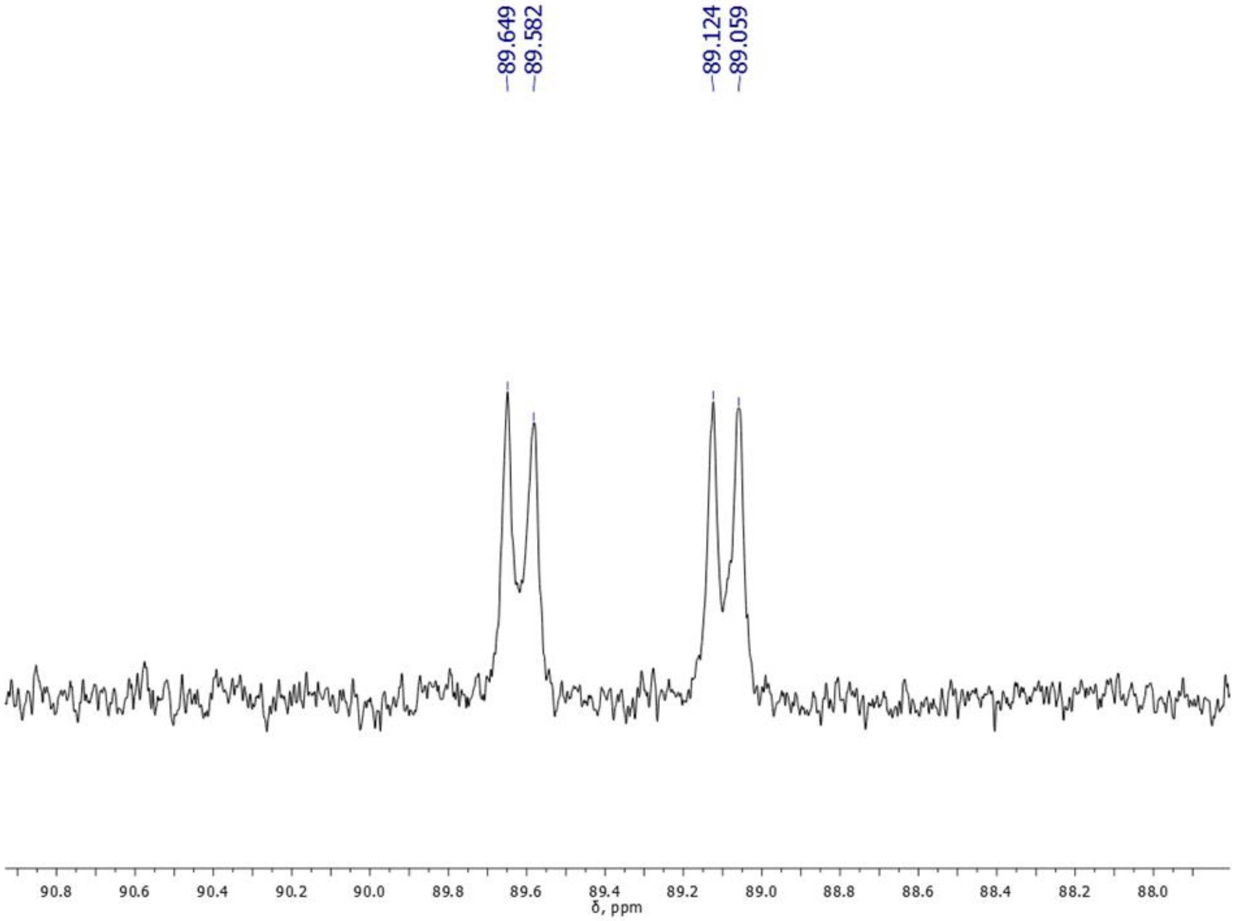

Figure S14: $^{31}P\{^{1}H\}$ NMR spectrum of [(PNP)Rh≡CF][$CHB_{11}Cl_{11}$] in C_6D_6/1,2-difluorobenzene.

Determination of $J_{C\text{-}F}$ by observation of ^{13}C satellites in ^{19}F NMR for [(PNP)Rh≡CF]$^+$. In an oven dried, argon cooled J. Young Tube [Ph_3C][$BArF_{20}$] (19 mg, 0.029 mmol) was dissolved in ~0.1 mL of C_6H_5F. To the solution Et_3SiH (6 mg, 0.052 mmol) was added and then mixed until the solution became a very pale brown (almost colorless). After five minutes, (PNP)Rh=CF_2 (17 mg, 0.029 mmol) was added and the NMR tube walls were rinsed with ~0.3 mL of C_6H_5F and ~0.2 mL of C_6D_6. Upon mixing the color immediately changed from a dark red to a dark blue. An overnight ^{19}F NMR collection was sufficient to show ^{13}C satellites (shown below). ^{19}F NMR (C_6H_5F/C_6D_6): 65.1(dt, $J_{Rh\text{-}F}$= 136 Hz, $J_{P\text{-}F}$= 12 Hz, $J_{C\text{-}F}$= 470 Hz)

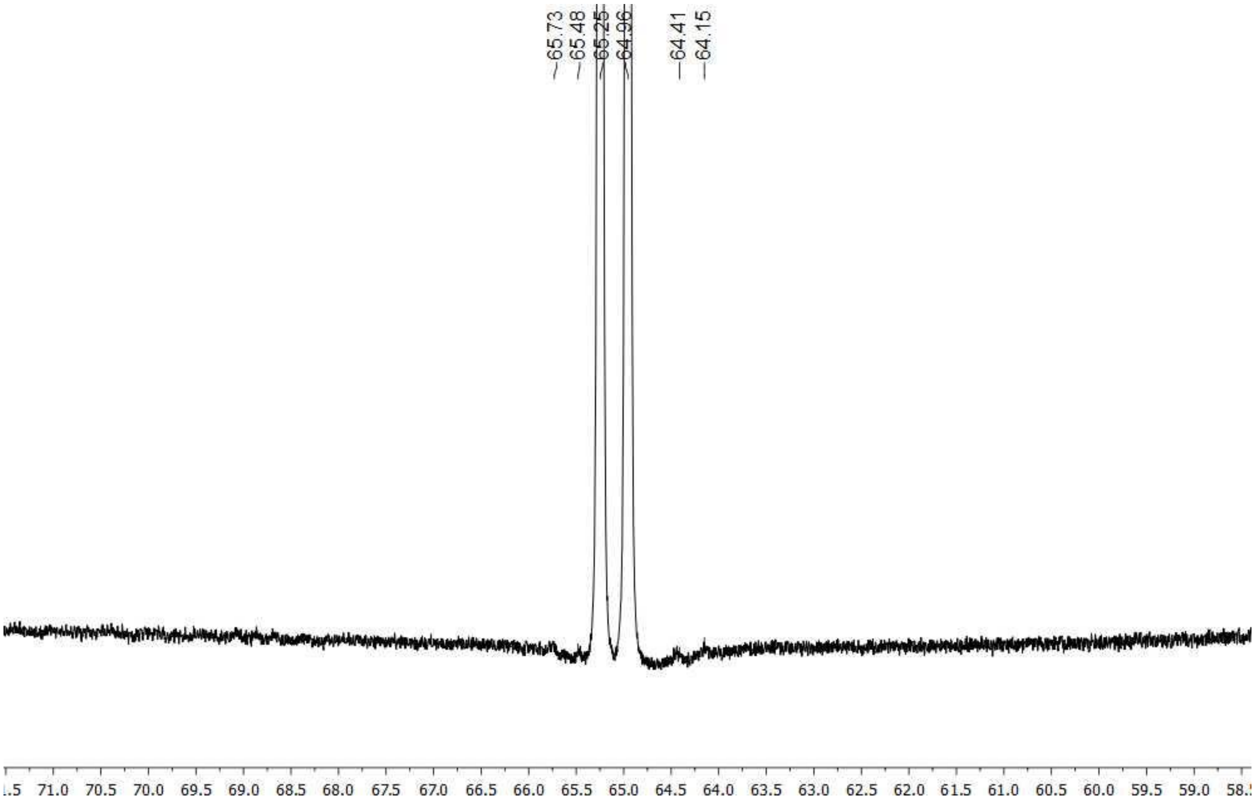

Figure S15: ^{19}F NMR spectrum of [(PNP)Rh≡CF][BArF$_{20}$] processed with a default line broadening factor of 0.88. ^{13}C NMR satellites are visible.

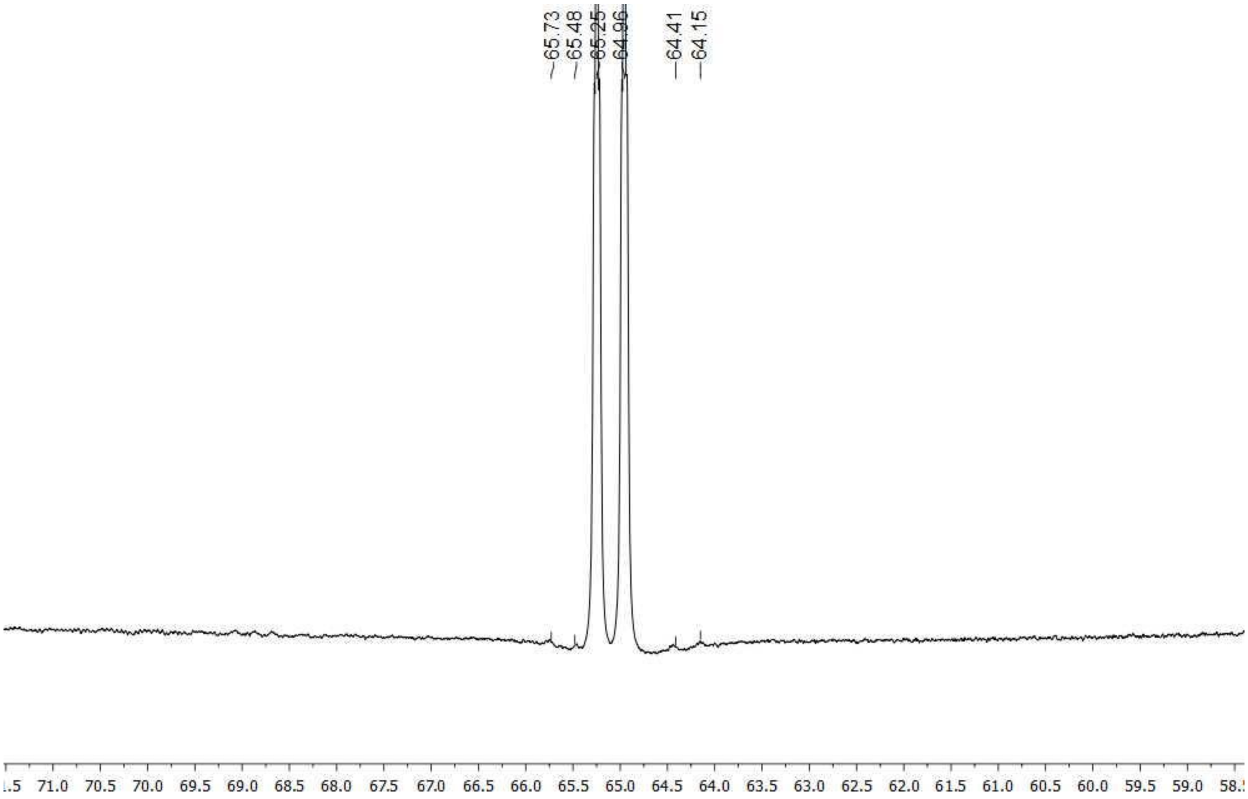

Figure S16: ^{19}F NMR of [(PNP)Rh≡CF][$BArF_{20}$] processed with a line broadening factor of 3.0 to improve the signal to noise ratio to better observe the ^{13}C satellites.

Treatment of (PNP)Rh(C_2F_4) with [($Et_3Si)_2(SO_3CF_3$)][$HCB_{11}Cl_{11}$]. [($Et_3Si)_2(SO_3CF_3$)][$HCB_{11}Cl_{11}$] (38 mg, 0.042 mmol) was dissolved in fluorobenzene in a vial and (PNP)Rh(C_2F_4) (27 mg, 0.042 mmol) was added to the reaction mixture, which immediately turned blue. The mixture was stirred overnight and transferred to a J. Young tube. Analysis by ^{19}F NMR spectroscopy showed Et_3SiOTf and Et_3SiF, while analysis by $^{31}P\{^1H\}$ NMR showed no signals. The reaction mixture was transferred back to a flask, and the volatiles were removed under vacuum. The dark blue solid was then redissolved in fluorobenzene and precipitated with pentane. A slightly blue-tinted clear solution was decanted from the blue solid, and the solid was dried under vacuum to yield 25 mg of a blue solid. The solid was dissolved in 0.5 mL of toluene and 0.2 mL of *ortho*-dichlorobenzene and transferred to a J. Young tube. Analysis by ^{19}F NMR spectroscopy showed a broad signal at -84.5 ppm. Upon cooling the solution to -30 °C this signal

split into two other signals at -83.2 and -85.8 ppm, and other broad signals appeared at -97.6 and -106.1 ppm. Analysis by $^{31}P\{^1H\}$ NMR spectroscopy showed one broad peak at 55 ppm, but upon cooling the sample to -30 °C the signal split into two broad peaks around 60 ppm.

The mixture was warmed to room temperature and CsF (10 mg, 0.66 mmol) was added to the J. Young tube and rotated overnight. By morning the solution had turned from dark blue to a brownish green. Analysis by ^{19}F NMR spectroscopy showed that 98% of the visible signals had been converted back to (PNP)Rh(C_2F_4) (as judged by integration versus a residual FPh peak). The solution in the J. Young tube was transferred to a vial and pentane was added to precipitate any cationic species and the solution was filtered through celite to form an orange filtrate. ^1H, $^{31}P\{^1H\}$, and ^{19}F NMR spectroscopy all confirmed that (PNP)Rh(C_2F_4) was the resulting product.

Treatment of (PNP)Rh=C(F)(CF_3) with [(Et_3Si)$_2$(SO_3CF_3)][$HCB_{11}Cl_{11}$]. A mixture of (PNP)Rh=C(F)(CF_3) and (PNP)Rh(CO) (21 mg, 9:1) was dissolved in fluorobenzene in a J. Young tube and treated with [(Et_3Si)$_2$(SO_3CF_3)][$HCB_{11}Cl_{11}$] (30 mg, 0.033 mmol). Analysis by ^{19}F NMR spectroscopy after 2 h at RT showed Et_3SiOTf and Et_3SiF. Analysis by $^{31}P\{^1H\}$ NMR showed a broad doublet at about 60 ppm. The reaction was left overnight and in the morning the volatiles were removed and the resulting solid was washed with pentane. The resulting solid was dissolved in C_6D_5Br for NMR analysis. Analysis by $^{31}P\{^1H\}$ NMR spectroscopy showed a broad doublet at 60.3 ppm (d, J = 148 Hz) as the major signal. Analysis by ^{19}F NMR spectroscopy showed a singlet at -71.8 ppm as the major signal. The ^1H NMR spectrum showed broad aliphatic peaks and no characteristic signals. This mixture was transferred to a 10 mL Schlenk flask and the volatiles were removed under vacuum. The solid was then dissolved in *ortho*-difluorobenzene and treated with CsF (5 mg, 0.033 mmol) and stirred for 4 days at room

temperature. The volatiles were removed and the resulting solid was dissolved in fluorobenzene and pentane and filtered through Celite. The volatiles were removed and the solid was dissolved in C_6D_6 for NMR analysis. Analysis by ^{19}F NMR spectroscopy showed (PNP)Rh=C(F)(CF_3) as the major fluorine containing product. Analysis by ^{31}P{^{1}H} NMR spectroscopy showed (PNP)Rh(CO), (PNP)Rh=C(F)(CF_3), and (PNP)Rh(C_6D_5)(Br) in a (20:51:28 ratio). ^{1}H NMR spectroscopy verified the identity and composition of this mixture. Method 2. A mixture of (PNP)Rh=C(F)(CF_3) and (PNP)Rh(CO) (11 mg, 1:1) was dissolved in fluorobenzene in a J. Young tube and treated with [$(Et_3Si)_2(SO_3CF_3)$][$HCB_{11}Cl_{11}$] (15 mg, 0.017 mmol). The reaction was left overnight and in the morning the volatiles were removed and the reaction mixture was dissolved in *ortho*-difluorobenzene and treated with CsF (5 mg, 0.033 mmol). The reaction was stirred for 4 days at room temperature. The volatiles were removed under vacuum and the resulting solid was dissolved in fluorobenzene. Pentane was added to the solution to precipitate any cesium salts, and the supernatant was passed through Celite. The volatiles were removed under vacuum, and the resulting solid was dissolved in C_6D_6 for NMR analysis. Analysis by ^{31}P{^{1}H} NMR showed (PNP)Rh(CO) and (PNP)Rh=C(F)(CF_3). ^{1}H NMR spectroscopy showed that these products were present in a ((PNP)Rh(CO) : (PNP)Rh=C(F)(CF_3) = 3:1 ratio). ^{19}F NMR spectroscopy showed (PNP)Rh=C(F)(CF_3) as the major fluorine-containing product. (PNP)Rh(C_2F_4) was not observed.

III. X-ray Crystallography

X-Ray data collection, solution, and refinement for (PNP)Rh=CF_2. A red block crystal of suitable size (0.30 x 0.21 x 0.15 mm) was selected from a representative sample of crystals of the same habit using an optical microscope, mounted onto a nylon loop and placed in a cold stream of nitrogen. Low temperature (110 K) X-ray data were obtained on a Bruker APEXII CCD based diffractometer (Mo sealed X-ray tube, K_α = 0.71073 Å). All diffractometer manipulations, including data collection, integration and scaling were carried out using the Bruker APEXII software.[Error! Bookmark not defined.] An absorption correction was applied using SADABS.[Error! Bookmark not defined.] The structure was solved in the $Pna2_1$ space group using XS[Error! Bookmark not defined.] (incorporated in SHELXTL). The solution was refined by full-matrix least squares on F^2. No additional symmetry was found using ADDSYMM incorporated into the PLATON program.[Error! Bookmark not defined.] All non-hydrogen atoms were refined with anisotropic thermal parameters. All hydrogen atoms were placed in idealized positions and refined using a riding model. The structure was refined (weighted least squares refinement on F^2) and the final least-squares refinement converged to R_1 = 0.0357 ($I > 2\sigma(I)$, 6606 data) and wR_2 = 0.0751 (F^2, 30909 data, 298 parameters).

X-Ray data collection, solution, and refinement for (PNP)Rh(C_2F_4).A red block crystal of suitable size (0.17 x 0.14 x 0.11 mm) was selected from a representative sample of crystals of the same habit using an optical microscope, mounted onto a nylon loop and placed in a cold stream of nitrogen. Low temperature (110 K) X-ray data were obtained on a Bruker APEXII CCD based diffractometer (Mo sealed X-ray tube, K_α = 0.71073 Å). All diffractometer manipulations, including data collection, integration and scaling were carried out using the Bruker APEXII software.[4] An absorption correction was applied using SADABS.[5] The structure was solved in the monoclinic C2/c space group using XS[6] (incorporated in SHELXTL). The

solution was refined by full-matrix least squares on F^2. No additional symmetry was found using ADDSYMM incorporated into the PLATON program.[7] All non-hydrogen atoms were refined with anisotropic thermal parameters. All hydrogen atoms were placed in idealized positions and refined using a riding model. The structure was refined (weighted least squares refinement on F^2) and the final least-squares refinement converged to $R_1 = 0.0293$ ($I > 2\sigma(I)$, 2775 data) and $wR_2 = 0.0862$ (F^2, 3031 data, 169 parameters).

X-Ray data collection, solution, and refinement for [(PNP)Rh≡CF][$CHB_{11}Cl_{11}$]. A blue prism (0.20 x 0.17 x 1.00 mm) was selected from a representative sample of crystals of the same habit using an optical microscope, mounted onto a nylon loop and placed in a cold stream of nitrogen (110 K). Low-temperature X-ray data were obtained on a Bruker APEXII CCD based diffractometer (Mo sealed X-ray tube, Kα = 0.71073 Å). All diffractometer manipulations, including data collection, integration and scaling were carried out using the Bruker APEXII software.**Error! Bookmark not defined.** The space group was determined on the basis of systematic absences and intensity statistics and the structure was solved in the orthorhombic space group $Pna2_1$ by direct methods using XS**Error! Bookmark not defined.** (incorporated in SHELXTL) and refined by full-matrix least-squares on F^2. No obvious missed symmetry was reported by PLATON.**Error! Bookmark not defined.** All non-hydrogen atoms were refined with anisotropic thermal parameters. Hydrogen atoms were placed in idealized positions and refined using riding model. The structure was refined (weighted least squares refinement on F^2) and the final least-squares refinement converged to $R_1 = 0.0590$ ($I > 2\sigma(I)$, 9513 data) and $wR_2 = 0.1511$ (F^2, 10849 data, 511 parameters).

IV. Computational Methods

Full molecule DFT studies were performed using the M06 functional[8,9] and the triple-ζ LACV3P**++ basis set, which used extended core potentials[10-13]on Rh and a 6-311G**++ basis[14-17] for other atoms, as implemented in the Jaguar[18,19] suite of programs. NBO 6.0[20-25] and WBI calculations,[26] as implemented in Jaguar, were performed on each optimized structure. Computed structures were confirmed as energy minima by calculating the vibrational frequencies using second derivative analytic methods, and confirming the absence of imaginary frequencies. DFT calculations on the energetics of rotation barriers in CX_2 and C_2X_4 ligands (X = F, H) were performed on complexes containing a truncated form of the pincer ligand (see text of manuscript).

Non-default options chosen in Jaguar:

- SCF calculation type: DFT(M06)
- Vibrational frequencies and related properties will be computed from analytic second derivatives
- Molecular symmetry not used
- Energy convergence criterion: 1.00E-05 hartrees
- RMS density matrix convergence criterion: 1.00E-06
- Highest accuracy cutoffs used in SCF

V. Natural Localized Molecular Orbitals for (PNP)Rh=CH_2

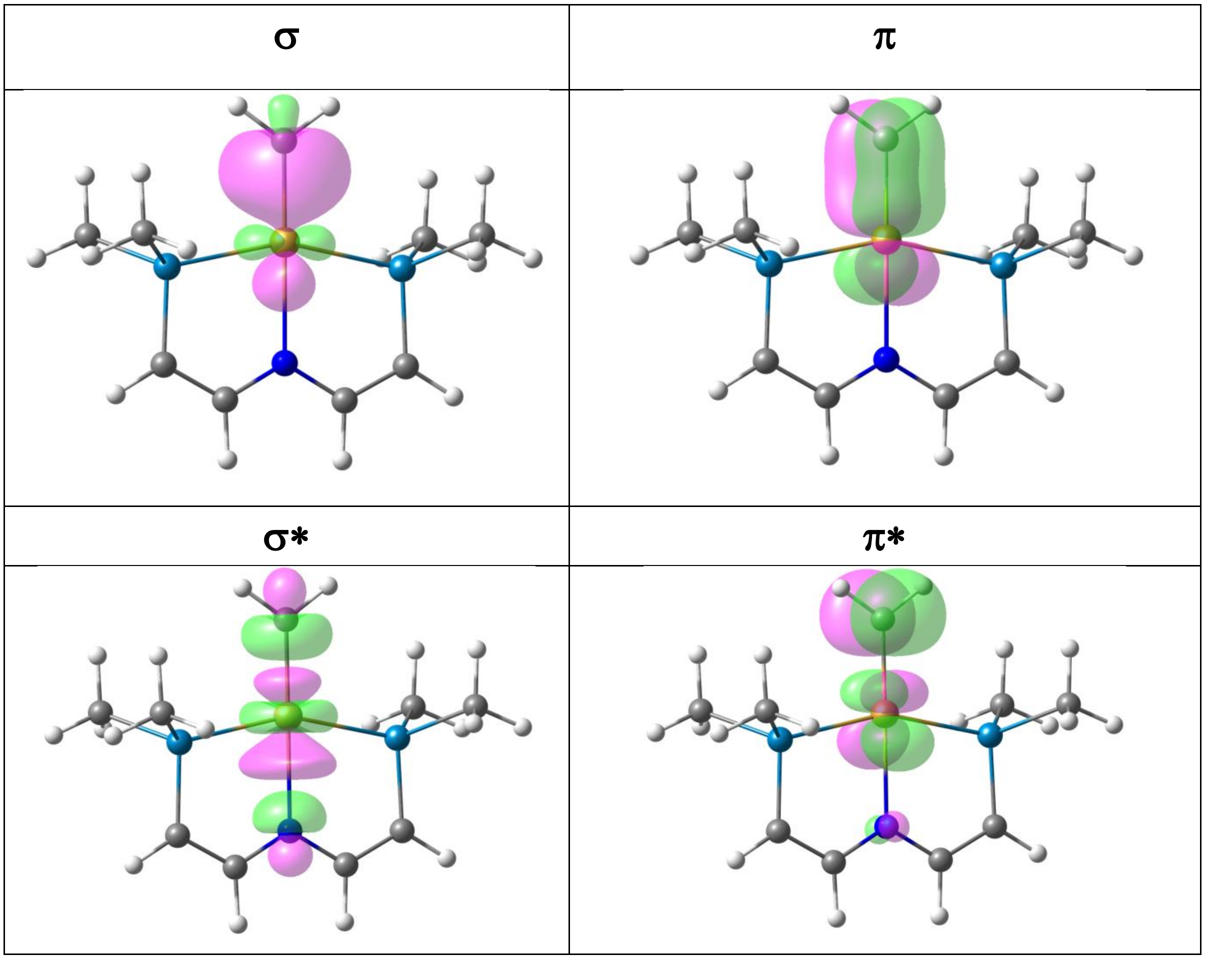

Figure S17: NLMOs for the bonding interactions between the CH_2 ligand and the **(PNP)Rh** fragment. For clarity the P^iPr_2 groups have been replaced by PMe_2 groups and the aryl part of the pincer truncated to P-CH=CH-N linkers.

VI. Cartesian Coordinates

(PNP)Rh(C_2H_4)

```
76
Rh(PINCER)(C2H4)
  Rh    6.898477   10.107683    7.593911
   P    7.154633    7.855462    7.045758
   P    7.355532   12.325380    8.151822
   N    8.993928   10.007310    7.618990
   C    9.609416    8.779485    7.805715
   C    8.897287    7.597916    7.507772
   C    9.477385    6.344166    7.690190
   H    8.902124    5.448707    7.457819
   C   10.759125    6.190300    8.196926
   C   11.439398    7.357987    8.548331
   H   12.427135    7.277142    8.999947
   C   10.892744    8.611329    8.364572
   H   11.450466    9.482741    8.689922
   C    9.728109   11.170676    7.448714
   C   11.034486   11.214903    6.920681
   H   11.514095   10.293797    6.607978
   C   11.702215   12.410115    6.750333
   H   12.703322   12.395348    6.321619
   C   11.128344   13.637992    7.086323
   C    9.826184   13.607778    7.562836
   H    9.333265   14.554305    7.781980
   C    9.125123   12.415237    7.730648
   C   11.380211    4.842460    8.397102
   H   11.690266    4.691350    9.437771
   H   12.274525    4.712699    7.776136
   H   10.682012    4.040485    8.139727
   C   11.880162   14.919932    6.901711
   H   12.763915   14.967445    7.549000
   H   11.254389   15.786340    7.135621
   H   12.234233   15.034819    5.870552
   C    7.445874    6.046646    4.853748
   H    7.296530    5.884657    3.779583
   H    6.890251    5.265516    5.383903
   H    8.513486    5.907996    5.059363
   C    7.004111    7.449487    5.234354
   H    5.931394    7.564876    5.018198
   C    7.768809    8.504064    4.445518
   H    8.841439    8.454196    4.669973
   H    7.427431    9.516020    4.692123
   H    7.643565    8.340882    3.368866
   C    4.779994    6.461062    7.518728
   H    4.252368    5.677700    8.074835
   H    4.654264    6.247532    6.452172
   H    4.274091    7.409300    7.734953
   C    6.242645    6.505070    7.938815
   H    6.715359    5.549472    7.670458
```

C	6.383660	6.716572	9.440069
H	5.965401	7.685197	9.738928
H	7.430440	6.698917	9.758049
H	5.846902	5.932515	9.986637
C	7.846724	11.621591	10.764134
H	8.924165	11.570199	10.564394
H	7.417287	10.646291	10.507617
H	7.712701	11.795899	11.837977
C	7.203017	12.743488	9.960301
H	6.119589	12.729742	10.151944
C	7.766096	14.098531	10.353498
H	7.608985	14.273839	11.424456
H	7.298127	14.928430	9.812901
H	8.846281	14.136243	10.171633
C	5.135899	13.939614	7.628026
H	4.698398	14.770178	7.062284
H	5.006170	14.163199	8.692030
H	4.547269	13.044049	7.397059
C	6.597136	13.756924	7.241710
H	7.152160	14.663115	7.523536
C	6.752000	13.533892	5.743512
H	6.250096	12.609879	5.432409
H	7.802721	13.451642	5.449623
H	6.305335	14.365996	5.187017
C	4.859063	10.194956	6.872945
C	4.843616	10.216190	8.266312
H	4.712661	11.109107	6.300556
H	4.656904	11.144113	8.802921
H	4.597320	9.320846	8.834084
H	4.597488	9.288460	6.330991

(PNP)Rh(C_2F_4)

76

Rh(PNP)(C2F4)

Rh	6.872524	10.112374	7.598432
P	7.182854	7.813792	8.124187
P	7.387384	12.371228	7.060831
N	8.969449	10.011744	7.570254
C	9.620699	9.030999	8.308348
C	8.934890	7.844671	8.628427
C	9.556707	6.838322	9.364590
H	9.010583	5.926610	9.601236
C	10.852655	6.968947	9.842463
C	11.505539	8.173389	9.576829
H	12.504564	8.332517	9.979468
C	10.916816	9.177558	8.835322
H	11.454019	10.107044	8.677717
C	9.690459	10.924977	6.810769
C	10.949987	10.653760	6.245949
H	11.399204	9.676619	6.390089
C	11.610746	11.596151	5.484403

H	12.576969	11.340375	5.052384
C	11.070206	12.858460	5.235196
C	9.807818	13.114026	5.751187
H	9.345505	14.074368	5.528454
C	9.113522	12.172233	6.507818
C	11.519289	5.887800	10.635659
H	11.865906	6.257368	11.607559
H	12.397266	5.488374	10.114611
H	10.838516	5.052199	10.821911
C	11.814859	13.869517	4.419140
H	12.744411	14.178906	4.911134
H	11.215060	14.768984	4.252791
H	12.091890	13.468422	3.437466
C	7.627256	5.320961	6.839646
H	7.521201	4.734439	5.919687
H	7.101931	4.780731	7.634027
H	8.694464	5.347939	7.088814
C	7.087982	6.724651	6.618692
H	6.014985	6.679129	6.388231
C	7.803185	7.411293	5.462394
H	8.871844	7.535327	5.678477
H	7.388489	8.405052	5.259941
H	7.713713	6.806104	4.553223
C	4.903325	6.515317	9.154740
H	4.482213	5.914469	9.968687
H	4.813347	5.931271	8.233351
H	4.285651	7.410277	9.049240
C	6.345836	6.869090	9.485115
H	6.914929	5.932873	9.577665
C	6.453916	7.633875	10.797437
H	5.917368	8.584977	10.740364
H	7.493258	7.844013	11.067378
H	6.007058	7.045296	11.606848
C	8.115391	12.712420	9.703678
H	9.160787	12.485677	9.459073
H	7.612299	11.763500	9.920107
H	8.109622	13.323629	10.613205
C	7.438674	13.464720	8.565188
H	6.381650	13.613550	8.824256
C	8.104670	14.809770	8.325936
H	8.080348	15.404040	9.246714
H	7.612890	15.397948	7.544229
H	9.157253	14.679815	8.048581
C	5.214581	13.881557	6.090508
H	4.829538	14.518666	5.286335
H	5.207271	14.472932	7.011609
H	4.517588	13.049867	6.217348
C	6.606790	13.391320	5.720899
H	7.259954	14.268820	5.609801
C	6.604744	12.619439	4.408291
H	5.982191	11.723580	4.482994
H	7.611503	12.311606	4.110035

```
   H    6.193190   13.247656    3.610026
   C    4.930822    9.918014    6.977058
   C    4.975988   10.490018    8.272122
   F    4.514886   10.663724    5.939549
   F    4.602346   11.773048    8.442755
   F    4.520292    9.785601    9.321702
   F    4.432414    8.676484    6.818442
```

(PNP)Rh=CH_2

```
73
Rh(PNP)(CH2)
  Rh    6.811662   10.087833    7.760333
   P    7.202815    7.900116    7.035527
   P    7.406014   12.303656    8.218492
   N    9.024316    9.976300    7.717989
   C    4.963417   10.153882    7.813060
   C    9.626839    8.755305    7.914361
   C    8.888098    7.584690    7.629947
   C    9.426150    6.317790    7.841773
   H    8.829692    5.433968    7.615868
   C   10.701586    6.142715    8.358726
   C   11.421924    7.299384    8.669325
   H   12.411580    7.198450    9.112983
   C   10.914616    8.564670    8.456120
   H   11.500359    9.430093    8.749119
   C    9.733950   11.112302    7.423979
   C   11.025326   11.136445    6.851144
   H   11.518878   10.199218    6.615676
   C   11.652145   12.324330    6.539774
   H   12.638861   12.291237    6.079428
   C   11.057327   13.568238    6.770729
   C    9.778634   13.558094    7.306592
   H    9.273275   14.511120    7.461235
   C    9.117471   12.371542    7.619297
   C   11.281503    4.783379    8.601646
   H   11.573935    4.650790    9.649963
   H   12.179498    4.611939    7.996215
   H   10.563912    3.994613    8.356675
   C   11.766778   14.840891    6.424273
   H   12.675453   14.974904    7.023416
   H   11.128263   15.712547    6.596763
   H   12.074012   14.858588    5.371913
   C    7.687591    6.331883    4.723061
   H    7.679381    6.283380    3.627838
   H    7.042198    5.527746    5.092556
   H    8.712992    6.126203    5.051960
   C    7.243428    7.709475    5.186301
   H    6.204118    7.893285    4.875942
   C    8.124940    8.802124    4.595035
   H    9.168446    8.673628    4.908259
   H    7.804400    9.799503    4.916898
```

H	8.096276	8.756780	3.500349
C	4.762842	6.572373	7.055857
H	4.117380	5.810993	7.508020
H	4.773604	6.399545	5.975152
H	4.295881	7.549703	7.237268
C	6.151415	6.515307	7.672879
H	6.636244	5.573718	7.377648
C	6.085541	6.591330	9.190878
H	5.613998	7.529541	9.506428
H	7.078568	6.550161	9.648352
H	5.490125	5.760496	9.586247
C	8.221371	11.797380	10.794518
H	9.275430	11.808919	10.491195
H	7.842137	10.782602	10.635663
H	8.178070	12.026902	11.865295
C	7.424546	12.824985	10.002575
H	6.369413	12.763149	10.307957
C	7.949521	14.231228	10.240132
H	7.905188	14.471825	11.308876
H	7.377416	14.997930	9.706696
H	8.998472	14.309418	9.931724
C	5.094316	13.813166	7.865757
H	4.535102	14.537871	7.263242
H	5.100561	14.175305	8.898717
H	4.538244	12.866964	7.842969
C	6.493748	13.630343	7.300523
H	7.058320	14.564500	7.432216
C	6.454100	13.275782	5.821142
H	5.918857	12.330459	5.668982
H	7.458440	13.158451	5.401712
H	5.935512	14.059445	5.256890
H	4.284947	9.297520	7.656865
H	4.389004	11.073275	8.020351

(PNP)Rh=CF$_2$

73
Rh(PNP)(CF2)

Rh	4.134653	15.994255	1.637800
P	2.603092	15.156823	0.083458
P	5.434650	16.316862	3.553488
N	3.391705	14.371660	2.786454
C	4.789961	17.423492	0.625333
C	2.186147	13.788619	2.426935
C	1.685416	13.973798	1.118430
C	0.476574	13.401708	0.723046
H	0.114431	13.564076	-0.291210
C	-0.310153	12.659395	1.589631
C	0.143453	12.548996	2.905422
H	-0.477346	12.033164	3.636411
C	1.341984	13.092262	3.317814

H	1.619182	13.013915	4.362581
C	4.127519	13.923732	3.872814
C	4.055706	12.615895	4.397794
H	3.408503	11.886880	3.924255
C	4.827698	12.221945	5.470422
H	4.744437	11.196398	5.827169
C	5.728137	13.085938	6.096669
C	5.858802	14.350817	5.545142
H	6.604228	15.023168	5.967812
C	5.101245	14.769056	4.451366
C	-1.599068	12.032125	1.157751
H	-2.416629	12.279047	1.844204
H	-1.526499	10.938391	1.128703
H	-1.890826	12.367139	0.158027
C	6.530850	12.644947	7.281118
H	5.903214	12.523339	8.172137
H	7.311591	13.370699	7.527603
H	7.017508	11.679917	7.100228
C	2.405022	13.475823	-2.214370
H	2.946425	12.992043	-3.035793
H	1.651569	14.133526	-2.660627
H	1.887276	12.686920	-1.656931
C	3.382371	14.216397	-1.316968
H	3.901206	14.993861	-1.896631
C	4.424095	13.269930	-0.734977
H	3.948095	12.512064	-0.100868
H	5.155935	13.805058	-0.119579
H	4.955902	12.749144	-1.539272
C	1.817988	17.170413	-1.711228
H	0.993661	17.734533	-2.162082
H	2.351554	16.660483	-2.519592
H	2.504050	17.895214	-1.261989
C	1.272147	16.214924	-0.661940
H	0.555087	15.532958	-1.140447
C	0.564978	16.976504	0.450449
H	1.271792	17.634184	0.972188
H	0.119975	16.306480	1.192523
H	-0.232300	17.601706	0.032248
C	3.367049	17.654890	4.769587
H	3.079170	16.747041	5.313625
H	2.865536	17.630903	3.795659
H	2.995266	18.517224	5.334471
C	4.880454	17.734840	4.618689
H	5.125791	18.623429	4.018804
C	5.565518	17.826167	5.971993
H	5.213506	18.714889	6.508744
H	6.655532	17.899633	5.895395
H	5.321422	16.954824	6.590297
C	7.788009	17.717064	2.924528
H	8.883293	17.740203	2.953003
H	7.423559	18.585165	3.482985
H	7.484748	17.839276	1.880067

C	7.288359	16.395163	3.485606
H	7.648539	16.288201	4.518596
C	7.802364	15.225025	2.658311
H	7.421537	15.287172	1.630922
H	7.495271	14.259403	3.072042
H	8.897413	15.245749	2.614237
F	4.870204	17.522062	-0.695398
F	5.284185	18.575924	1.059027

[(PNP)Rh≡CH]$^+$

72

Rh(PNP)(CH)_CATION

Rh	6.885615	10.111489	7.582160
P	7.192419	7.819708	7.023196
P	7.398066	12.354679	8.184032
N	8.958106	10.013741	7.606195
C	5.159688	10.196379	7.553947
C	9.604088	8.785004	7.819204
C	8.915979	7.597417	7.520420
C	9.500953	6.351331	7.725093
H	8.943765	5.445419	7.492428
C	10.778227	6.229921	8.259084
C	11.431097	7.410184	8.614655
H	12.409069	7.349024	9.086599
C	10.869622	8.656150	8.405850
H	11.407127	9.540240	8.730314
C	9.720755	11.178062	7.416207
C	11.003889	11.191309	6.853907
H	11.462005	10.262705	6.531971
C	11.682728	12.380404	6.665926
H	12.671015	12.353599	6.212468
C	11.136271	13.614522	7.020097
C	9.844666	13.609797	7.531572
H	9.371287	14.562011	7.764886
C	9.142113	12.422000	7.714695
C	11.424372	4.896514	8.461892
H	11.965624	4.855895	9.411863
H	12.151920	4.686689	7.669335
H	10.690416	4.086482	8.454972
C	11.907965	14.881529	6.830709
H	12.723814	14.958995	7.558106
H	11.273669	15.763336	6.952594
H	12.361522	14.926259	5.835700
C	7.397496	6.050300	4.849743
H	7.241124	5.893410	3.777471
H	6.802444	5.300060	5.379799
H	8.457452	5.862066	5.053931
C	7.021645	7.478349	5.214435
H	5.955506	7.650899	5.007332
C	7.847275	8.491457	4.432194
H	8.914325	8.390557	4.663662

H	7.549776	9.522796	4.649356
H	7.723698	8.318378	3.358521
C	4.748164	6.594483	7.512459
H	4.169428	5.879017	8.105083
H	4.627385	6.324339	6.459252
H	4.294311	7.581855	7.669079
C	6.198194	6.586075	7.966289
H	6.641879	5.606106	7.737827
C	6.326357	6.858437	9.458398
H	5.893112	7.833617	9.712204
H	7.367533	6.850851	9.794781
H	5.783277	6.094502	10.023707
C	7.949239	11.554502	10.764543
H	9.023974	11.555320	10.546762
H	7.555128	10.562416	10.518813
H	7.829605	11.713355	11.840908
C	7.236659	12.662851	9.999952
H	6.156140	12.589866	10.190805
C	7.744840	14.037808	10.406110
H	7.592394	14.181891	11.480780
H	7.231151	14.855505	9.890872
H	8.820234	14.127563	10.215875
C	5.088668	13.820785	7.705962
H	4.587238	14.602223	7.126389
H	4.981698	14.075142	8.764486
H	4.545088	12.885215	7.520134
C	6.538231	13.702623	7.265946
H	7.070587	14.628973	7.526377
C	6.657438	13.460523	5.768019
H	6.135974	12.539034	5.482130
H	7.698284	13.377233	5.441350
H	6.196691	14.288503	5.220075
H	4.061410	10.251976	7.532489

[(PNP)Rh≡CF]$^{+}$

72

Rh(PNP)(CF) CATION

Rh	6.881593	10.119588	7.514502
P	7.205545	7.817103	6.993520
P	7.402042	12.348552	8.181176
N	8.939323	10.027821	7.587007
C	5.154490	10.219387	7.327304
C	9.578338	8.806487	7.847833
C	8.908778	7.610071	7.554878
C	9.495336	6.370555	7.802847
H	8.950932	5.456330	7.572876
C	10.755616	6.270333	8.374673
C	11.393311	7.464143	8.717756
H	12.360959	7.418815	9.212602
C	10.830577	8.701190	8.468507
H	11.356012	9.597983	8.778713

C	9.700267	11.187534	7.369561
C	10.982437	11.187468	6.806761
H	11.440191	10.251607	6.504825
C	11.660948	12.373722	6.599640
H	12.648919	12.339441	6.145664
C	11.115590	13.614686	6.933306
C	9.828662	13.619990	7.454344
H	9.359412	14.575571	7.682322
C	9.127688	12.434028	7.658007
C	11.399400	4.947886	8.647384
H	11.692540	4.856503	9.698355
H	12.308214	4.818829	8.049773
H	10.728361	4.117818	8.412878
C	11.884522	14.877821	6.707103
H	12.765336	14.926983	7.356561
H	11.275246	15.762458	6.908657
H	12.244338	14.945242	5.675246
C	7.461826	5.984008	4.878999
H	7.345229	5.800791	3.805868
H	6.836823	5.255836	5.404844
H	8.510465	5.788246	5.128974
C	7.092380	7.426270	5.190974
H	6.037278	7.606120	4.937976
C	7.962779	8.405828	4.414979
H	9.018902	8.288388	4.684805
H	7.681171	9.447325	4.600606
H	7.871154	8.212136	3.341691
C	4.749333	6.583675	7.445846
H	4.171516	5.854878	8.022918
H	4.663930	6.308748	6.390223
H	4.265324	7.557639	7.589431
C	6.185444	6.609021	7.941708
H	6.645809	5.626703	7.760902
C	6.265019	6.933018	9.427181
H	5.820276	7.914412	9.633508
H	7.295230	6.942017	9.796270
H	5.706875	6.187157	10.001759
C	8.056196	11.407220	10.692563
H	9.121448	11.418831	10.432569
H	7.652288	10.428186	10.410521
H	7.979357	11.508862	11.779780
C	7.316724	12.556129	10.017099
H	6.245257	12.478337	10.251890
C	7.853903	13.905653	10.470635
H	7.757064	13.992491	11.557965
H	7.321082	14.752670	10.026699
H	8.918866	14.000849	10.230054
C	5.095400	13.879578	7.919518
H	4.575545	14.692037	7.402505
H	5.060546	14.095577	8.990977
H	4.518453	12.964810	7.737382
C	6.512518	13.753043	7.384847

H	7.080519	14.653965	7.659120
C	6.536749	13.592286	5.871978
H	5.962719	12.710153	5.564234
H	7.552797	13.488115	5.480077
H	6.079710	14.466595	5.397952
F	3.930797	10.305611	7.103783

(PNP)Rh(CO)

72

Rh(PNP)(CO)

Rh	6.862922	10.109381	7.595235
P	7.160986	7.866182	7.016251
P	7.365464	12.316322	8.173344
N	8.977439	10.008944	7.606261
C	5.010252	10.196483	7.586282
C	9.584032	8.792903	7.859644
C	8.859802	7.605177	7.616624
C	9.414645	6.356923	7.886283
H	8.831488	5.456646	7.695183
C	10.686852	6.219229	8.423603
C	11.385800	7.395905	8.699979
H	12.369565	7.327239	9.161931
C	10.863087	8.644714	8.430048
H	11.432598	9.528948	8.697315
C	9.698796	11.161541	7.357649
C	10.991467	11.186761	6.799419
H	11.476881	10.252044	6.538006
C	11.633203	12.379782	6.533983
H	12.623282	12.353464	6.081129
C	11.046949	13.618078	6.803360
C	9.761899	13.602691	7.327710
H	9.264577	14.554533	7.511837
C	9.088152	12.413164	7.592225
C	11.283610	4.877946	8.719655
H	11.622304	4.808108	9.759733
H	12.155090	4.674022	8.086128
H	10.560467	4.074498	8.551626
C	11.772695	14.895892	6.514385
H	12.644187	15.022678	7.167783
H	11.124316	15.764906	6.660062
H	12.141147	14.925218	5.482599
C	7.636031	6.118415	4.829354
H	7.608450	5.982964	3.741797
H	7.002964	5.342913	5.273407
H	8.668694	5.945306	5.154105
C	7.190585	7.526137	5.188786
H	6.148956	7.681451	4.871214
C	8.065194	8.572244	4.509326
H	9.109041	8.477845	4.833776
H	7.736982	9.590572	4.744347
H	8.039455	8.436866	3.422104

```
   C    4.742976    6.501851    7.164326
   H    4.125359    5.751552    7.670496
   H    4.755608    6.253951    6.098433
   H    4.241402    7.469564    7.281254
   C    6.135163    6.527809    7.778405
   H    6.635517    5.570562    7.573832
   C    6.069527    6.745981    9.283293
   H    5.569573    7.695073    9.510541
   H    7.064182    6.774423    9.739368
   H    5.497939    5.940440    9.758112
   C    8.167837   11.539931   10.693473
   H    9.219764   11.533206   10.381723
   H    7.746992   10.556490   10.457755
   H    8.141946   11.681965   11.779845
   C    7.405115   12.661382    9.999656
   H    6.349644   12.607325   10.304672
   C    7.978191   14.021886   10.360096
   H    7.950418   14.163979   11.446804
   H    7.427295   14.852212    9.905795
   H    9.026548   14.094515   10.047876
   C    5.087050   13.898575    7.985682
   H    4.549447   14.701437    7.468950
   H    5.106424   14.147353    9.051258
   H    4.499642   12.981237    7.862544
   C    6.479951   13.741635    7.393101
   H    7.064000   14.648893    7.603576
   C    6.417146   13.525768    5.887774
   H    5.835248   12.626107    5.654514
   H    7.411729   13.404382    5.447031
   H    5.929494   14.379121    5.402932
   O    3.859207   10.250087    7.580856
```

[(PNP)Rh(NO)]$^{+}$

```
72
Rh(PNP)(NO)CATION
  Rh    6.870663   10.108861    7.596229
   P    7.185259    7.821336    6.969913
   P    7.393720   12.358976    8.219700
   N    8.887622   10.013091    7.606645
   N    5.095108   10.192047    7.586578
   C    9.533749    8.793900    7.877236
   C    8.869579    7.596290    7.581801
   C    9.455853    6.361868    7.846073
   H    8.918918    5.443695    7.614594
   C   10.711251    6.272269    8.433872
   C   11.346788    7.469574    8.766730
   H   12.311432    7.430248    9.267587
   C   10.783293    8.703585    8.500640
   H   11.303795    9.606622    8.801646
   C    9.649109   11.164705    7.340511
   C   10.907445   11.134301    6.729185
```

H	11.342465	10.185145	6.434470
C	11.588589	12.308329	6.467025
H	12.557246	12.253925	5.975306
C	11.067057	13.561578	6.792219
C	9.802686	13.593276	7.367003
H	9.352723	14.558900	7.590970
C	9.098904	12.420903	7.627133
C	11.354414	4.954022	8.726625
H	11.670126	4.888949	9.772798
H	12.249486	4.805559	8.112870
H	10.675098	4.121431	8.527971
C	11.837249	14.811584	6.506350
H	12.723144	14.884901	7.146543
H	11.232463	15.706010	6.675159
H	12.189445	14.833458	5.470190
C	7.591861	6.118884	4.777087
H	7.532903	5.988252	3.691597
H	6.963236	5.348164	5.233683
H	8.632032	5.937364	5.070272
C	7.166738	7.533084	5.143714
H	6.119515	7.698070	4.851218
C	8.042487	8.574068	4.458173
H	9.088861	8.473609	4.770631
H	7.721397	9.597659	4.678384
H	8.005962	8.429934	3.373806
C	4.739319	6.510989	7.170010
H	4.124366	5.772318	7.693584
H	4.746134	6.240017	6.110521
H	4.232558	7.477716	7.271721
C	6.131072	6.545129	7.783236
H	6.629567	5.582507	7.598411
C	6.082948	6.800478	9.282845
H	5.564935	7.742134	9.501173
H	7.080835	6.849773	9.729615
H	5.530076	5.996987	9.779520
C	8.148387	11.546515	10.745370
H	9.203274	11.548920	10.445657
H	7.737491	10.555480	10.525847
H	8.112070	11.699598	11.828521
C	7.380095	12.659262	10.043960
H	6.318743	12.592775	10.324218
C	7.928619	14.030770	10.408900
H	7.868705	14.172876	11.492895
H	7.378869	14.853016	9.940552
H	8.984526	14.114408	10.128018
C	5.084175	13.891529	7.981180
H	4.547549	14.681028	7.445776
H	5.102990	14.167448	9.039243
H	4.490649	12.975671	7.877899
C	6.474272	13.723635	7.386541
H	7.058394	14.636430	7.573299
C	6.421647	13.464868	5.887642

H	5.820537	12.574506	5.666966
H	7.416229	13.319638	5.454516
H	5.952959	14.313550	5.379666
O	3.955632	10.244573	7.579137

(PNP)Rh=C(F)(CF_3)

76

Rh(PNP)(CFCF3)

Rh	6.841979	10.242011	7.611036
P	7.180984	8.043141	6.874918
P	7.442921	12.480730	7.977646
N	9.021902	10.086992	7.600322
C	5.006317	10.361844	7.725465
C	9.599004	8.848808	7.801475
C	8.849501	7.694006	7.492353
C	9.362670	6.416690	7.703787
H	8.757513	5.544687	7.457594
C	10.623489	6.218361	8.246514
C	11.352839	7.360952	8.585171
H	12.329719	7.241183	9.051273
C	10.868969	8.635733	8.372763
H	11.460203	9.489062	8.688420
C	9.767348	11.204611	7.308174
C	11.073529	11.184102	6.770700
H	11.546660	10.231117	6.559209
C	11.744545	12.348892	6.465174
H	12.742356	12.280260	6.033889
C	11.181624	13.612662	6.665146
C	9.886447	13.646020	7.157346
H	9.402501	14.614358	7.280460
C	9.181320	12.482347	7.462824
C	11.178246	4.848559	8.488066
H	11.453111	4.704802	9.539463
H	12.082037	4.667741	7.894367
H	10.451919	4.073359	8.227049
C	11.940857	14.858902	6.329022
H	12.827055	14.976806	6.963916
H	11.321505	15.751085	6.460480
H	12.292187	14.848985	5.290630
C	7.662615	6.536940	4.531472
H	7.673547	6.518630	3.435582
H	6.989377	5.741134	4.866670
H	8.676084	6.295158	4.872885
C	7.247372	7.914412	5.022634
H	6.218447	8.135394	4.702486
C	8.169330	8.995204	4.473243
H	9.202373	8.834476	4.805567
H	7.866183	9.994566	4.803808
H	8.162872	8.969491	3.377695
C	4.725400	6.747198	6.800210
H	4.064214	5.979382	7.216620

```
   H    4.764720    6.599567    5.716653
   H    4.254624    7.718587    6.991497
   C    6.096612    6.660987    7.453418
   H    6.581278    5.723596    7.144612
   C    5.995002    6.691880    8.970990
   H    5.520953    7.619235    9.310696
   H    6.975433    6.624364    9.451800
   H    5.381479    5.855181    9.323220
   C    8.096806   12.037531   10.608015
   H    9.166503   12.024557   10.366146
   H    7.708985   11.024300   10.460973
   H    7.994745   12.301841   11.666387
   C    7.363874   13.052243    9.741288
   H    6.291839   13.015122    9.985990
   C    7.896204   14.458237    9.963910
   H    7.789119   14.734027   11.019327
   H    7.369929   15.214117    9.371551
   H    8.963561   14.510992    9.719968
   C    5.182675   14.016629    7.413860
   H    4.716748   14.790507    6.793677
   H    5.120135   14.345723    8.455897
   H    4.577842   13.109532    7.307712
   C    6.610299   13.775615    6.950305
   H    7.190297   14.701585    7.073789
   C    6.647662   13.353003    5.488390
   H    6.093155   12.416702    5.348840
   H    7.670142   13.196278    5.130437
   H    6.179743   14.121215    4.862345
   F    4.117010   10.514199    6.728642
   C    4.162697   10.323902    9.001029
   F    3.279791    9.317543    8.961205
   F    4.911425   10.162260   10.084462
   F    3.474919   11.463929    9.148766
```

[(PNP)Rh≡CCF$_3$]$^+$

```
75
Rh(PNP)(CCF3) CATION
  Rh    6.835781   10.088918    7.389219
   P    7.171578    7.820635    6.727130
   P    7.316120   12.359958    7.920165
   N    8.887614    9.984662    7.392457
   C    5.143597   10.201293    7.748598
   C    9.525125    8.762876    7.672498
   C    8.857172    7.575364    7.335854
   C    9.426361    6.332997    7.587857
   H    8.889218    5.424027    7.323399
   C   10.664965    6.220290    8.210783
   C   11.297407    7.403153    8.593529
   H   12.243987    7.344581    9.125779
   C   10.751018    8.647332    8.336931
   H   11.267372    9.536929    8.680593
```

C	9.656862	11.151137	7.235787
C	10.959372	11.147091	6.718832
H	11.423389	10.209588	6.432689
C	11.646563	12.329978	6.527555
H	12.649386	12.291687	6.108219
C	11.090656	13.572437	6.836642
C	9.785868	13.581228	7.311132
H	9.309866	14.538777	7.513374
C	9.072935	12.398758	7.496549
C	11.285848	4.890402	8.495237
H	11.534822	4.787798	9.556437
H	12.218025	4.761532	7.934857
H	10.619199	4.068036	8.224895
C	11.866646	14.833833	6.630342
H	12.737452	14.874730	7.293341
H	11.257538	15.719423	6.827021
H	12.242849	14.904527	5.604652
C	7.582630	6.210528	4.472218
H	7.520097	6.119598	3.382951
H	6.961390	5.418705	4.901940
H	8.625400	6.026524	4.754805
C	7.146872	7.606702	4.891600
H	6.095207	7.772331	4.617130
C	8.004687	8.680574	4.235109
H	9.054609	8.586851	4.537676
H	7.668481	9.692169	4.487139
H	7.962936	8.569114	3.147111
C	4.718513	6.536956	6.887178
H	4.077799	5.827016	7.419376
H	4.710638	6.260295	5.829200
H	4.254200	7.525242	6.985727
C	6.114460	6.517368	7.489557
H	6.602055	5.563909	7.238070
C	6.093664	6.679505	9.002371
H	5.604032	7.616601	9.289130
H	7.098298	6.673027	9.435852
H	5.525590	5.860113	9.453741
C	7.734780	11.703764	10.562739
H	8.814584	11.641847	10.380542
H	7.299974	10.717090	10.371512
H	7.591167	11.940023	11.621639
C	7.094719	12.786040	9.704127
H	6.006727	12.762871	9.854832
C	7.632738	14.167686	10.044989
H	7.427951	14.386701	11.097958
H	7.174652	14.965100	9.451192
H	8.719119	14.210529	9.909778
C	5.022519	13.783768	7.256108
H	4.557599	14.549375	6.627289
H	4.878081	14.083168	8.298570
H	4.471193	12.851842	7.080270
C	6.485158	13.628849	6.874874

H	7.014974	14.571836	7.074013
C	6.649396	13.257748	5.407486
H	6.129166	12.316938	5.189992
H	7.699727	13.144146	5.120935
H	6.209351	14.035203	4.775281
C	3.844229	10.323256	8.485643
F	4.007561	11.058075	9.585530
F	2.913353	10.900266	7.736034
F	3.425674	9.111597	8.836512

[(PNP)Rh(η^1-CFCF$_2$)]$^+$

75
Rh(PNP)(h1-CFCF2) CATION

Rh	6.980467	10.192671	7.687308
P	7.113171	7.967718	6.979790
P	7.381583	12.475476	8.165332
N	8.983941	10.074534	7.668764
C	5.009183	10.302030	7.349125
C	9.614657	8.823174	7.742260
C	8.866314	7.671812	7.425281
C	9.439455	6.418077	7.560163
H	8.864668	5.530807	7.305165
C	10.736251	6.250545	8.046872
C	11.443774	7.395282	8.419873
H	12.431893	7.287681	8.859480
C	10.909039	8.655918	8.265996
H	11.466032	9.515976	8.618860
C	9.750740	11.219420	7.448647
C	11.008832	11.189464	6.817026
H	11.410707	10.249385	6.457635
C	11.709684	12.352694	6.591635
H	12.671060	12.307748	6.086725
C	11.198003	13.594195	6.976469
C	9.915327	13.628081	7.529685
H	9.484520	14.599028	7.760228
C	9.178703	12.476453	7.748688
C	11.344639	4.897378	8.199151
H	11.696187	4.739152	9.224006
H	12.216602	4.785164	7.545470
H	10.636270	4.103160	7.954826
C	11.987024	14.839944	6.757733
H	12.873069	14.854894	7.402471
H	11.402615	15.737416	6.970055
H	12.348528	14.899587	5.726015
C	7.415396	6.379213	4.659716
H	7.260416	6.305393	3.578133
H	6.846886	5.567969	5.124893
H	8.482211	6.208185	4.845069
C	6.989900	7.756892	5.140953
H	5.920586	7.910532	4.942312
C	7.774282	8.861149	4.445988

H	8.848178	8.781906	4.660290
H	7.434776	9.856631	4.753210
H	7.651458	8.777261	3.361417
C	4.723394	6.572154	7.334442
H	4.211198	5.728386	7.807343
H	4.608449	6.464068	6.252143
H	4.203784	7.486143	7.638593
C	6.177776	6.591020	7.783102
H	6.659841	5.654787	7.468005
C	6.283509	6.733229	9.295341
H	5.809807	7.664975	9.626772
H	7.319610	6.735865	9.648291
H	5.763710	5.903370	9.784626
C	7.744453	11.981751	10.845641
H	8.817807	11.812182	10.692295
H	7.223460	11.030874	10.690308
H	7.605221	12.282258	11.888967
C	7.223287	13.067192	9.915386
H	6.137808	13.154468	10.054249
C	7.876691	14.411337	10.190484
H	7.660664	14.717874	11.219197
H	7.512283	15.206975	9.532917
H	8.966390	14.349581	10.095657
C	5.283518	14.149805	7.357491
H	4.959702	14.971392	6.710311
H	5.125556	14.464697	8.393187
H	4.629135	13.296322	7.149654
C	6.733368	13.807661	7.049212
H	7.352416	14.697330	7.230385
C	6.895007	13.370587	5.599078
H	6.282924	12.483995	5.394824
H	7.933483	13.134588	5.341507
H	6.558994	14.169540	4.929795
F	4.437027	9.590017	6.342394
C	4.089664	10.856319	8.165685
F	4.391873	11.599993	9.203126
F	2.802312	10.671111	8.084517

[(PNP)Rh(η^2-CFCF$_2$)]$^+$

75
Rh(PNP)(h2-CFCF2)

Rh	6.856493	10.113526	7.659430
P	7.215289	7.835527	6.993516
P	7.427123	12.389906	8.152059
N	8.946278	10.005800	7.687894
C	9.592809	8.770045	7.872054
C	8.914481	7.592502	7.540571
C	9.495431	6.340031	7.722415
H	8.942031	5.439378	7.460281
C	10.764916	6.210446	8.261245

C	11.420077	7.385270	8.636896
H	12.397731	7.312242	9.109208
C	10.859374	8.633452	8.450300
H	11.397667	9.514757	8.784526
C	9.708241	11.142991	7.398673
C	10.975622	11.111334	6.795107
H	11.420842	10.160997	6.524226
C	11.656367	12.280497	6.511919
H	12.631240	12.215989	6.033497
C	11.129796	13.541825	6.798715
C	9.857579	13.580633	7.351751
H	9.401563	14.550020	7.539026
C	9.154541	12.411042	7.635118
C	11.403941	4.873027	8.468049
H	11.700206	4.727499	9.509633
H	12.312054	4.765027	7.856456
H	10.723970	4.055638	8.192697
C	11.891842	14.785418	6.482495
H	12.815875	14.849593	7.072793
H	11.307225	15.685247	6.690029
H	12.189183	14.814975	5.426596
C	7.610245	6.399234	4.622024
H	7.540085	6.387179	3.529685
H	7.003823	5.567440	4.996468
H	8.657278	6.217048	4.885672
C	7.150620	7.753867	5.142038
H	6.092067	7.911384	4.892705
C	7.975791	8.886284	4.542629
H	9.027841	8.807937	4.837281
H	7.613727	9.878968	4.849219
H	7.929725	8.832160	3.445881
C	4.780734	6.489314	7.150561
H	4.216583	5.650581	7.568320
H	4.708907	6.422354	6.059843
H	4.272075	7.404066	7.478347
C	6.216488	6.435378	7.654922
H	6.698428	5.539202	7.233613
C	6.297511	6.366615	9.173288
H	5.847713	7.248576	9.637673
H	7.330566	6.284403	9.529685
H	5.750107	5.489083	9.528378
C	7.962991	11.763748	10.780120
H	9.026077	11.662992	10.532272
H	7.487318	10.787474	10.632879
H	7.890070	12.024584	11.839065
C	7.307309	12.849720	9.937174
H	6.229455	12.871042	10.153000
C	7.912686	14.219333	10.214247
H	7.793918	14.457853	11.275543
H	7.435198	15.021249	9.643227
H	8.988504	14.223771	9.996417
C	5.137752	13.913517	7.549926

H	4.721797	14.733987	6.955274
H	5.037210	14.179683	8.601657
H	4.517380	13.032492	7.356908
C	6.580443	13.674220	7.127914
H	7.159139	14.589336	7.310159
C	6.683871	13.309006	5.652144
H	6.107372	12.402547	5.436624
H	7.716199	13.142894	5.331153
H	6.268408	14.116514	5.044756
C	5.085487	10.179296	7.147161
C	4.956939	10.176344	8.542601
F	4.298170	10.091858	6.131173
F	4.626649	11.261771	9.239936
F	4.524872	9.103650	9.202402

34

Rh_PINCER_STRIPPED_C2F4 PERP

Rh	6.902212	10.110491	7.567406
P	7.177114	7.836202	6.955697
P	7.376712	12.347672	8.193817
N	9.004232	10.010076	7.594403
C	9.631440	8.838103	7.289584
C	8.964339	7.709429	6.964548
C	9.732291	11.117574	7.915697
C	9.167676	12.304764	8.226101
C	6.629298	7.249128	5.312753
H	5.538692	7.178988	5.291131
C	6.564081	6.509894	8.055532
H	7.002609	5.548702	7.766267
C	6.849837	12.975532	9.828642
H	5.770693	13.149372	9.826741
C	6.916765	13.731623	7.090476
H	7.436195	14.645870	7.397474
C	5.001965	10.388574	6.856260
C	4.963043	10.017899	8.220072
F	4.651650	11.632803	6.495619
F	4.574972	10.913903	9.140894
F	4.482659	8.813785	8.567316
F	4.558301	9.532865	5.922482
H	6.840467	6.738990	9.087171
H	5.474642	6.450131	7.989699
H	6.949551	7.961001	4.548700
H	7.064501	6.266624	5.100194
H	9.483400	6.786338	6.724655
H	10.725875	8.844822	7.318707
H	10.821535	11.007021	7.912828
H	9.766129	13.174701	8.479362
H	7.194764	13.483613	6.063679
H	5.836427	13.892802	7.131766
H	7.083376	12.232082	10.594070
H	7.371858	13.910713	10.058456

34

Rh_PINCER_STRIPPED_C2F4_45_DEG

Rh	6.931047	10.134277	7.753177
P	7.238621	7.846088	7.255127
P	7.462668	12.413414	8.097932
N	9.054069	10.054287	7.628146
C	9.680195	8.879701	7.348034
C	9.014932	7.720570	7.151577
C	9.794569	11.182406	7.799390
C	9.245219	12.395103	8.027650
C	6.568797	7.284340	5.650094
H	5.476837	7.287078	5.681041
C	6.688449	6.542832	8.411876
H	7.090865	5.572881	8.099731

C	7.011299	13.165600	9.700902
H	5.927128	13.274953	9.773130
C	6.897328	13.657873	6.884582
H	7.391262	14.617267	7.073342
C	4.990228	10.829419	7.512940
C	4.975122	9.594388	8.191257
F	4.544585	11.926781	8.160777
F	4.673455	9.566332	9.495031
F	4.381612	8.538703	7.593158
F	4.566541	10.885856	6.243689
H	7.055700	6.777772	9.413483
H	5.597267	6.493428	8.433850
H	6.905802	7.969433	4.868942
H	6.927969	6.274083	5.425106
H	9.526331	6.787546	6.937317
H	10.772637	8.909058	7.289224
H	10.881327	11.068760	7.737626
H	9.847288	13.288322	8.160863
H	7.151599	13.314156	5.879209
H	5.814315	13.786595	6.954296
H	7.361022	12.516355	10.506512
H	7.486160	14.148268	9.797807

34

Rh_PINCER_STRIPPED_C2F4_INPLANE

Rh	6.889890	10.113098	7.603849
P	7.237443	7.842178	7.017334
P	7.427262	12.395516	7.941253
N	9.025949	10.030313	7.580627
C	9.665692	8.857732	7.334982
C	9.013028	7.707264	7.063109
C	9.760345	11.146648	7.824018
C	9.204662	12.356767	8.048852
C	6.714341	7.354867	5.334756
H	5.624638	7.299960	5.279366
C	6.582585	6.477399	8.038458
H	7.061341	5.539013	7.738032
C	6.824230	13.322925	9.393273
H	5.754536	13.523693	9.294886
C	7.017782	13.520355	6.558761
H	7.512512	14.485929	6.710332
C	4.969379	10.855987	7.897480
C	4.905159	9.491019	7.555382
F	4.636409	11.216797	9.147674
F	4.496765	8.617397	8.491815
F	4.367902	9.142729	6.373474
F	4.475294	11.765679	7.038150
H	6.796634	6.673905	9.091079
H	5.500994	6.393979	7.908452
H	7.066941	8.105069	4.623434
H	7.146162	6.380908	5.080275

H	9.531925	6.773322	6.872085
H	10.759125	8.880927	7.365323
H	10.847727	11.025980	7.829253
H	9.797602	13.244637	8.243696
H	7.372911	13.075228	5.626574
H	5.937271	13.666021	6.494386
H	6.989900	12.733957	10.297698
H	7.367874	14.270814	9.470052

34

Rh_PINCER_STRIPPED_C2H4_PERP

Rh	6.942365	10.108465	7.568047
P	7.193123	7.866675	6.958703
P	7.390160	12.316835	8.186175
N	9.027892	10.008962	7.594110
C	9.651077	8.838611	7.287169
C	8.978471	7.709613	6.960897
C	9.751873	11.115619	7.915674
C	9.181877	12.303949	8.226398
C	6.629818	7.279070	5.313167
H	5.535680	7.243485	5.283894
C	6.560201	6.526697	8.042292
H	6.955410	5.553090	7.731638
C	6.845934	12.955569	9.819068
H	5.759583	13.093744	9.822734
C	6.912782	13.710378	7.090537
H	7.391089	14.642201	7.412291
C	4.911879	10.390992	6.868986
C	4.874888	10.025280	8.209500
H	4.774480	11.428223	6.570218
H	4.707999	10.769237	8.985785
H	4.628479	9.006781	8.502661
H	4.694884	9.665773	6.087348
H	6.865320	6.727678	9.071994
H	5.466430	6.495915	7.998145
H	6.979833	7.975568	4.547546
H	7.023110	6.279002	5.099198
H	9.495708	6.786082	6.717511
H	10.745988	8.842226	7.314578
H	10.841854	11.008207	7.913933
H	9.778483	13.174760	8.482175
H	7.221627	13.481119	6.067872
H	5.826127	13.844833	7.109142
H	7.110844	12.229568	10.591424
H	7.326249	13.914363	10.044033

34

Rh_PINCER_STRIPPED_45_DEG

Rh	6.953387	10.124232	7.459174
P	7.218505	7.878054	6.892345
P	7.436010	12.319322	8.079250
N	9.040498	9.975190	7.634070
C	9.655178	8.780552	7.426095
C	8.983219	7.657399	7.078156
C	9.775187	11.075069	7.948184
C	9.220316	12.286451	8.191081
C	6.795348	7.200677	5.240111
H	5.710348	7.192008	5.096546
C	6.395718	6.664640	7.994750
H	6.701746	5.638889	7.760202
C	6.845709	13.091617	9.635630
H	5.764597	13.257171	9.594657
C	6.986668	13.606638	6.851894
H	7.387360	14.586932	7.133496
C	4.943408	10.021588	6.642443
C	4.872159	10.517219	7.938648
H	4.893942	10.693732	5.787028
H	4.687817	11.575379	8.109122
H	4.591892	9.868171	8.766940
H	4.642712	8.997710	6.432765
H	6.659547	6.894186	9.029827
H	5.308813	6.748260	7.883246
H	7.247664	7.830295	4.470508
H	7.174355	6.177754	5.136799
H	9.491301	6.713121	6.907604
H	10.743009	8.759878	7.549798
H	10.860583	10.941175	8.003967
H	9.821460	13.152324	8.451411
H	7.386738	13.317445	5.877214
H	5.895428	13.673791	6.776954
H	7.068837	12.425290	10.471965
H	7.345026	14.053269	9.798683

34

Rh_PINCER_STRIPPED_C2H4_INPLANE

Rh	6.916306	10.105350	7.580047
P	7.236235	7.855262	7.051971
P	7.430754	12.267750	8.296310
N	9.032609	10.042758	7.467971
C	9.667094	8.891478	7.131583
C	9.006634	7.738961	6.871005
C	9.767560	11.138173	7.785704
C	9.211168	12.301840	8.196273
C	6.506225	7.099575	5.550275
H	5.420343	7.016023	5.659790
C	6.766770	6.622995	8.327398
H	7.167000	5.636284	8.068695
C	6.991661	12.743694	10.011824
H	5.909208	12.879373	10.101632
C	6.839925	13.734195	7.367252

H	7.345059	14.638994	7.723707
C	4.904474	10.820177	7.913783
C	4.843883	9.525272	7.400177
H	4.729243	10.992485	8.974130
H	4.602371	8.693474	8.059459
H	4.598469	9.371251	6.350859
H	4.686003	11.664381	7.262606
H	7.177000	6.937175	9.289852
H	5.677514	6.554577	8.410698
H	6.728672	7.730420	4.686582
H	6.924135	6.100439	5.383823
H	9.521761	6.822890	6.599055
H	10.759861	8.930114	7.081718
H	10.854488	11.041522	7.699424
H	9.807154	13.175360	8.442124
H	7.057689	13.596440	6.305651
H	5.760112	13.857448	7.496797
H	7.308076	11.950781	10.693398
H	7.491893	13.678581	10.288091

31

Rh_PINCER_STRIPPED_CF2_INPLANE

Rh	6.923821	10.109679	7.591744
P	7.168805	7.847727	7.023835
P	7.372115	12.338494	8.166363
N	9.017657	10.010683	7.608307
C	5.051452	10.198527	7.577502
C	9.635934	8.830050	7.324963
C	8.956558	7.697714	7.025676
C	9.739952	11.127190	7.905119
C	9.165760	12.318808	8.194352
C	6.591540	7.253057	5.390617
H	5.497438	7.266132	5.366504
C	6.528212	6.539651	8.133736
H	6.888864	5.553307	7.821576
C	6.827046	12.983078	9.791736
H	5.736338	13.073033	9.798611
C	6.876302	13.702619	7.049883
H	7.324251	14.649711	7.370416
F	4.283150	11.248286	7.828896
F	4.190929	9.226561	7.312425
H	7.272283	13.963782	9.992720
H	7.127692	12.282246	10.573680
H	5.786146	13.798309	7.055464
H	7.204969	13.473112	6.033901
H	5.434107	6.548164	8.111133
H	6.861384	6.739734	9.154468
H	6.945541	6.234252	5.197951
H	6.969052	7.920356	4.612693
H	9.761506	13.195864	8.429384

```
   H   10.830340   11.025123    7.905207
   H   10.730922    8.828271    7.344173
   H    9.470616    6.767794    6.801205
```

31

Rh_PINCER_STRIPPED_CF2_45DEG

```
  Rh    6.911330   10.116351    7.602412
   P    7.179943    7.863596    7.026185
   P    7.383352   12.366634    8.088129
   N    9.024389   10.020612    7.603599
   C    5.040864   10.144347    7.668776
   C    9.642356    8.841255    7.331797
   C    8.962313    7.702757    7.053047
   C    9.746392   11.140265    7.873283
   C    9.173740   12.342650    8.121754
   C    6.629952    7.382748    5.346339
   H    5.537282    7.405299    5.296323
   C    6.493980    6.500955    8.037339
   H    6.857734    5.530559    7.681774
   C    6.832932   13.027316    9.705042
   H    5.743898   13.132570    9.707777
   C    6.897459   13.719803    6.953909
   H    7.366445   14.662955    7.255839
   F    4.279178   10.720056    8.582812
   F    4.184934    9.513913    6.880074
   H    7.292392   14.002066    9.903462
   H    7.118859   12.325642   10.491809
   H    5.809830   13.839406    6.965304
   H    7.213241   13.469464    5.938801
   H    5.401056    6.518864    7.975117
   H    6.789471    6.637949    9.079756
   H    6.985175    6.377011    5.095481
   H    7.029952    8.097892    4.624043
   H    9.768221   13.226329    8.334230
   H   10.836744   11.037113    7.878465
   H   10.737445    8.840190    7.347772
   H    9.472708    6.768624    6.838692
```

31

Rh_PINCER_STRIPPED_CF2_PERP

```
  Rh    6.897614   10.113963    7.595079
   P    7.197896    7.860273    7.022702
   P    7.401106   12.333712    8.166699
   N    9.034621   10.013642    7.610110
   C    5.027248   10.184995    7.576884
   C    9.653854    8.842886    7.322474
   C    8.976845    7.708031    7.018040
   C    9.757605   11.120812    7.907933
```

C	9.186455	12.315265	8.200487
C	6.594824	7.288261	5.391833
H	5.500400	7.301065	5.379209
C	6.542914	6.566710	8.139938
H	6.897098	5.575172	7.836754
C	6.833176	12.970809	9.786295
H	5.742880	13.066327	9.782774
C	6.895316	13.682031	7.036457
H	7.342262	14.633804	7.344694
F	4.239543	10.452615	6.552097
F	4.199953	9.960409	8.580645
H	7.281154	13.947785	9.999474
H	7.120371	12.263134	10.567038
H	5.805129	13.777271	7.038996
H	7.224045	13.439807	6.023519
H	5.448734	6.581255	8.119950
H	6.877585	6.774386	9.158598
H	6.947249	6.272740	5.179671
H	6.961746	7.967518	4.619304
H	9.779293	13.193604	8.436971
H	10.847935	11.016820	7.906096
H	10.748958	8.842851	7.341932
H	9.487390	6.777517	6.789498

31

Rh_PINCER_STRIPPED_CH2_INPLANE

Rh	6.918454	10.106152	7.604752
P	7.160957	7.842887	7.036062
P	7.366239	12.335951	8.179367
N	9.023188	10.006758	7.617222
C	5.039043	10.195860	7.592629
C	9.638532	8.824882	7.327699
C	8.953111	7.695152	7.029170
C	9.743599	11.125465	7.915120
C	9.164103	12.314668	8.207186
C	6.587541	7.232243	5.404519
H	5.493026	7.245834	5.375120
C	6.540155	6.520871	8.144953
H	6.896307	5.535898	7.823166
C	6.833043	12.997403	9.804673
H	5.741783	13.086322	9.820916
C	6.884763	13.710129	7.064633
H	7.327137	14.657705	7.391690
H	4.357303	9.360037	7.364267
H	4.437398	11.093922	7.809534
H	6.965752	7.894328	4.622440
H	6.938647	6.211194	5.217944
H	6.884855	6.716123	9.162815
H	5.445209	6.525280	8.139468
H	7.221984	13.483546	6.050766

H	5.794168	13.807702	7.057002
H	7.138142	12.302729	10.590408
H	7.275907	13.981086	9.996672
H	9.759183	13.192990	8.440956
H	10.835317	11.031993	7.911524
H	10.734088	8.815191	7.344038
H	9.465568	6.764494	6.802224

31
Rh_PINCER_STRIPPED_CH2_45DEG

Rh	6.894013	10.106256	7.571755
P	7.204968	7.837461	7.043034
P	7.399857	12.315930	8.183601
N	9.048967	10.001777	7.610509
C	5.036517	10.220039	7.524066
C	9.668767	8.831295	7.320196
C	8.987906	7.694433	7.029645
C	9.767465	11.103423	7.940245
C	9.187717	12.293621	8.235959
C	6.618035	7.380670	5.367409
H	5.523554	7.410953	5.344342
C	6.578746	6.432706	8.038739
H	6.976690	5.485169	7.658083
C	6.819807	12.755140	9.866391
H	5.726789	12.824278	9.868658
C	6.928773	13.815259	7.241172
H	7.402719	14.704834	7.671408
H	4.407713	9.856611	6.694209
H	4.408786	10.708557	8.287764
H	7.004437	8.107095	4.648930
H	6.958330	6.376855	5.089381
H	6.887183	6.560253	9.078428
H	5.485813	6.406161	7.995979
H	9.497477	6.760924	6.809670
H	10.764607	8.826292	7.336371
H	10.858765	11.004250	7.957764
H	9.775660	13.171770	8.486315
H	7.247638	13.704971	6.202627
H	5.842139	13.941570	7.264785
H	7.122871	11.967555	10.559947
H	7.243531	13.711563	10.192681

31
Rh_PINCER_STRIPPED_CH2_PERP

Rh	6.855743	10.109023	7.605220
P	7.212397	7.859167	7.019602
P	7.417082	12.316898	8.195052
N	9.052928	10.005841	7.615123

C	5.008054	10.182484	7.597299
C	9.670781	8.841161	7.321024
C	8.988631	7.708905	7.010121
C	9.774874	11.104979	7.924490
C	9.199474	12.296388	8.229767
C	6.611367	7.284568	5.387371
H	5.516861	7.283380	5.378566
C	6.566168	6.548810	8.124518
H	6.938707	5.565281	7.816358
C	6.852570	12.947108	9.819865
H	5.763492	13.055690	9.813589
C	6.916765	13.684604	7.083798
H	7.380409	14.626322	7.398922
H	4.373565	9.996088	8.479347
H	4.394989	10.410660	6.709992
H	6.969508	7.969424	4.615761
H	6.977216	6.273902	5.173499
H	6.889762	6.753511	9.147259
H	5.472113	6.547957	8.093332
H	7.232366	13.449887	6.065012
H	5.827892	13.793404	7.100446
H	7.131129	12.230194	10.595171
H	7.312817	13.916958	10.040351
H	9.787360	13.175975	8.474413
H	10.866725	11.007550	7.922278
H	10.766770	8.833507	7.340244
H	9.493361	6.776837	6.774317

VII. References

1 M. Puri, S.Gatard, D. A. Smith and O. V. Ozerov, *Organometallics*, 2011, **30**, 2472.

2 L. P. Press, B. J. McCulloch, W. Gu, C.-H. Chen, B. M. Foxman and O. V. Ozerov, *Chem Commun.*, 2015, **51**, 14034.

3 S. Gatard, C. Guo, B. M. Foxman, O. V. Ozerov, *Organometallics*, 2007, **26**, 6066.

4 APEX2 "Program for Data Collection and Integration on Area Detectors" BRUKER AXS Inc., 5465 East Cheryl Parkway, Madison, WI 53711-5373 USA.

5 G. M. Sheldrick, "SADABS (version 2008/1): Program for Absorption Correction for Data from Area Detector Frames", University of Göttingen, Germany, 2008.

6 G. M. Sheldrick, *Acta Cryst.*, 2008, **A64**, 112.

7 A. L. Spek, (2006) PLATON – A Multipurpose Crystallographic Tool, Utrecht University, Utrecht, The Netherlands. A. L. Spek, *Acta Cryst*., 1990, **A46**, C34.

[8] Y. Zhao and D. G. Truhlar, *Theor. Chem. Acc.*, 2008, **120**, 215.

[9] Y. Zhao and D. G. Truhlar, *Acc. Chem. Res.* 2008, **41**, 157.

[10] W. R. Wadt and P. J. Hay, *J. Chem. Phys.*, 1985, **82**, 284.

[11] P. J. Hay and W. R. Wadt, *J. Chem. Phys.* 1985, **82**, 299.

[12] P. J. Hay and W. R. Wadt, *J. Chem. Phys.* 1985, **82**, 270.

[13] T. H. Dunning and P. J. Hay in *Modern Theoretical Chemistry, Vol. 4: Applications of Electronic Structure Theory*; ed. H. F. Schaefer III, Plenum, 1977, pp 461.

[14] M. J. Frisch, J. A. Pople and J. S. Binkley, *J. Chem. Phys.*, 1984, **80**, 3265.

[15] T. Clark, J. Chandrasekhar, G. W. Spitznagel and P. V. R. Schleyer, *J. Comput. Chem.*, 1983, **4**, 294.

[16] A. D. McLean and G. S. Chandler, *J. Chem. Phys.* 1980, **72**, 5639.

[17] R. Krishnan, J. S. Binkley, R. Seeger and J. A. Pople *J. Chem. Phys.* 1980, **72**, 650.

[18] A. D. Bochevarov, E. Harder, T. F. Hughes, J. R. Greenwood, D. A. Braden, D. M. Philipp, D. Rinaldo, M. D. Halls, J. Zhang and R. A. Friesner, *Int. J. Quantum Chem.* 2013, **113**, 2110.

[19] *Jaguar, versions 7.0-9.3*; Schrödinger, LLC, New York, NY, 2007-2016.

[20] C. R. Landis and F. Weinhold in *The Chemical Bond: Fundamental Aspects of Chemical Bonding*, ed. G. Frenking and S. Shaik, Wiley-VCH Verlag GmbH & Co. KGaA, Weinheim, 2014, vol. 1, pp 91-119.

[21] E. D. Glendening, J. K. Badenhoop, A. K. Reed, J. E. Carpenter, J. A. Bohmann, C. M. Morales, C. R. Landis and F. Weinhold, *NBO 6.0*, Theoretical Chemistry Institute, University of Wisconsin, Madison, WI, 2013.

[22] F. Weinhold and C. R. Landis, *Discovering Chemistry with Natural Bond Orbitals*; John Wiley & Sons, Hoboken, 2012.

[23] E. D. Glendening, C. R. Landis, and F. Weinhold, *Wiley Interdisciplinary Reviews: Computational Molecular Science*, 2012, **2**, 1.

[24] F. Weinhold and C. R. Landis, *Valency and Bonding: A Natural Bond Orbital Donor-Acceptor Perspective*, Cambridge University Press, Cambridge, 2005.

[25] A. E. Reed, L. A. Curtiss and F. Weinhold, *Chem. Rev.*, 1988, **88**, 899.

[26] K. B. Wiberg, *Tetrahedron*, 1968, **24**, 1083.
